# Biochemical, Anatomical, and Pharmacological Characterization of Calcitonin-Type Neuropeptides in Starfish: Discovery of an Ancient Role as Muscle Relaxants

**DOI:** 10.3389/fnins.2018.00382

**Published:** 2018-06-08

**Authors:** Weigang Cai, Chan-Hee Kim, Hye-Jin Go, Michaela Egertová, Cleidiane G. Zampronio, Alexandra M. Jones, Nam Gyu Park, Maurice R. Elphick

**Affiliations:** ^1^School of Biological & Chemical Sciences Queen Mary University of London, London, United Kingdom; ^2^Department of Biotechnology, College of Fisheries Sciences Pukyong National University, Busan, South Korea; ^3^School of Life Sciences and Proteomics Research Technology Platform University of Warwick, Coventry, United Kingdom

**Keywords:** calcitonin, echinoderm, *Asterias rubens*, starfish, evolution, neuropeptide

## Abstract

Calcitonin (CT) is a peptide hormone released by the thyroid gland that regulates blood Ca^2+^ levels in mammals. The CT gene is alternatively spliced, with one transcript encoding CT and another transcript encoding the CT-like neuropeptide calcitonin-gene related peptide (α-CGRP), which is a powerful vasodilator. Other CT-related peptides in vertebrates include adrenomedullin, amylin, and intermedin, which also act as smooth muscle relaxants. The evolutionary origin of CT-type peptides has been traced to the bilaterian common ancestor of protostomes and deuterostomes and a CT-like peptide (DH31) has been identified as a diuretic hormone in some insect species. However, little is known about the physiological roles of CT-type peptides in other invertebrates. Here we characterized a CT-type neuropeptide in a deuterostomian invertebrate—the starfish *Asterias rubens* (Phylum Echinodermata). A CT-type precursor cDNA (ArCTP) was sequenced and the predicted structure of the peptide (ArCT) derived from ArCTP was confirmed using mass spectrometry. The distribution of ArCTP mRNA and the ArCT peptide was investigated using *in situ* hybridization and immunohistochemistry, respectively, revealing stained cells/processes in the nervous system, digestive system, and muscular organs, including the apical muscle and tube feet. Investigation of the effects of synthetic ArCT on *in vitro* preparations of the apical muscle and tube feet revealed that it acts as a relaxant, causing dose-dependent reversal of acetylcholine-induced contraction. Furthermore, a muscle relaxant present in whole-animal extracts of another starfish species, *Patiria pectinifera*, was identified as an ortholog of ArCT and named PpCT. Consistent with the expression pattern of ArCTP in *A. rubens*, RT-qPCR revealed that in *P. pectinifera* the PpCT precursor transcript is more abundant in the radial nerve cords than in other tissues/organs analyzed. In conclusion, our findings indicate that the physiological action of CT-related peptides as muscle relaxants in vertebrates may reflect an evolutionarily ancient role of CT-type neuropeptides that can be traced back to the common ancestor of deuterostomes.

## Introduction

The thyroid hormone calcitonin was discovered in mammals as a regulator of blood calcium levels (Copp and Cameron, 1961; Copp et al., 1962) and identified as 32-residue C-terminally amidated peptide with an N-terminal disulfide bond (Potts et al., 1968; Niall et al., 1969). Sequencing of the gene encoding the calcitonin precursor revealed that it is alternatively spliced to produce two transcript types, one encoding calcitonin and another encoding a calcitonin-like peptide known as calcitonin gene-related peptide (αCGRP) (Amara et al., 1982; Morris et al., 1984). Investigation of the physiological roles of CGRP revealed that it is a neuropeptide that acts as a potent relaxant of vascular muscle (Brain et al., 1985). More recently, other peptides that are related to calcitonin and CGRP have been identified in mammals, including βCGRP, amylin, adrenomedullin, adrenomedullin 2 (intermedin), and calcitonin receptor-stimulating peptide (CRSP), and a shared characteristic of all of these peptides is the presence of an N-terminal disulfide bond (Steenbergh et al., 1985; Cooper et al., 1987; Kitamura et al., 1993; Katafuchi et al., 2003; Roh et al., 2004; Takei et al., 2004). Furthermore, all of these calcitonin-related peptides exert their physiological effects by binding to the calcitonin receptor (CTR) or CTR-like receptor (CLR), which are G-protein coupled receptors belonging to the secretin-type receptor family (Lin et al., 1991; Njuki et al., 1993; Hay et al., 2018).

Phylogenomic studies indicate that the evolutionary origin of calcitonin-type signaling can be traced back to the common ancestor of the Bilateria (Mirabeau and Joly, 2013). Thus, genes encoding calcitonin-like peptides and calcitonin receptor-like proteins have been identified in deuterostomian invertebrates, including the urochordate *Ciona intestinalis* (Sekiguchi et al., 2009), the cephalochordate *Branchiostoma floridae* (Sekiguchi et al., 2016), and the echinoderm *Strongylocentrotus purpuratus* (Rowe and Elphick, 2012), and in protostomian invertebrates, including insects [e.g., *Diploptera punctata* (Furuya et al., 2000; Zandawala, 2012)] and the mollusk *Lottia gigantea* (Veenstra, 2010). Furthermore, it appears that a gene duplication in a common ancestor of the protostomes gave rise to two types of calcitonin-related peptides. Firstly, calcitonin-like peptides that have a pair of N-terminally located cysteine residues and secondly calcitonin-like peptides without N-terminally located cysteine residues (Veenstra, 2011, 2014; Conzelmann et al., 2013; Jekely, 2013; Mirabeau and Joly, 2013). Nothing is known about the physiological roles of the cysteine-containing calcitonin-type peptides in protostomes, which may in part reflect the fact that genes encoding this peptide have been lost in some insect orders, including *Drosophila melanogaster* and other dipterans (Veenstra, 2014). However, protostomian calcitonin-like peptides without cysteine residues have been functionally characterized in insects and other arthropods as the 31-residue diuretic hormone named DH31 (Furuya et al., 2000; Coast et al., 2001; Te Brugge et al., 2009). Interestingly, DH31-type peptides are also present in annelids but they appear to have been lost in mollusks and nematodes (Conzelmann et al., 2013; Veenstra, 2014).

Turning to the deuterostomian invertebrates, calcitonin-type signaling has been characterized in the invertebrate chordates *C. intestinalis* (sub-phylum Urochordata) and *B. floridae* (sub-phylum Cephalochordata). In *C. intestinalis*, a single gene encoding a calcitonin-like peptide (CiCT) was identified and analysis of the expression of CiCT revealed that it is expressed in the neural complex, stigmata cells of the gill, blood cells, and endostyle, but the physiological roles of CiCT are not known (Sekiguchi et al., 2009). In *B. floridae*, three genes encoding calcitonin-like peptides (Bf-CTFPs) and one gene encoding a calcitonin receptor-like protein (Bf-CTFP-R) were identified. Furthermore, experimental studies showed that all three of the Bf-CTFPs act as ligands for Bf-CTFP-R, but only when the receptor is co-expressed with one of three *B. floridae* receptor activity-modifying proteins (RAMPs). Thus, this was the first study to demonstrate the existence of a functional calcitonin-type signaling system in a deuterostomian invertebrate (Sekiguchi et al., 2016). However, nothing is known about the physiological roles of Bf-CTFPs in *B. floridae*. Genes encoding calcitonin-type peptides and receptors have also been identified in ambulacrarian deuterostomes—hemichordates and echinoderms. For example, a calcitonin-type precursor and receptor was identified in the sea urchin *S. purpuratus* (Burke et al., 2006; Rowe and Elphick, 2012). However, nothing is known about the physiological roles of calcitonin-type signaling in hemichordates or echinoderms.

The aim of this study was to investigate the physiological roles of calcitonin-type peptides in echinoderms and to accomplish this we selected starfish as model experimental systems. Starfish (and other echinoderms) are important model systems for neuropeptide research because as non-chordate deuterostomes they occupy an “intermediate” evolutionary position with respect to vertebrates and the well-studied protostomian invertebrates (e.g., *D. melanogaster* and *Caenorhabditis elegans*). Therefore, echinoderms can provide key insights into the evolutionary history and comparative physiology of neuropeptide signaling systems (Semmens and Elphick, 2017). For example, identification of the receptor for the neuropeptide NGFFFamide in *S. purpuratus* enabled reconstruction of the evolution of a family of neuropeptides that include neuropeptide-S (NPS) in vertebrates and crustacean cardioactive peptide (CCAP)-type neuropeptides in protostomes (Semmens et al., 2015). Similarly, identification of peptide ligands for a gonadotropin-releasing hormone (GnRH)-type receptor and a corazonin-type receptor in the starfish *Asterias rubens* demonstrated that the evolutionary origin of these paralogous neuropeptide signaling systems can be traced to the common ancestor of the Bilateria (Tian et al., 2016). Furthermore, echinoderms typically exhibit pentaradial symmetry as adult animals, providing a unique context for comparative analysis of the physiological roles of neuropeptides in the Bilateria. For example, we recently reported detailed analyses of the distribution and actions of GnRH-type, corazonin-type, and pedal peptide/orcokinin-type neuropeptides in *A. rubens*, providing new insights into the evolution of neuropeptide function in the animal kingdom (Lin et al., 2017a; Tian et al., 2017).

Sequencing of the neural transcriptome of *A. rubens* has enabled identification of at least forty neuropeptide precursor proteins, including a calcitonin-type precursor named ArCTP (Semmens et al., 2016). Here we have confirmed the predicted structure of the peptide (ArCT) derived from ArCTP using mass spectrometry and we have investigated the distribution of the ArCTP transcript and the ArCT peptide in *A. rubens* using *in situ* hybridization and immunohistochemistry, respectively. Informed by the anatomical expression data, investigation of the *in vitro* pharmacological effects of synthetic ArCT revealed that it acts as muscle relaxant in *A. rubens*. Consistent with this finding, a muscle relaxant present in extracts of the starfish *Patiria pectinifera* was purified and identified as a calcitonin-type peptide. This is the first study to determine the physiological roles of calcitonin-type peptides in deuterostomian invertebrates. Furthermore, our findings indicate that the action of CT-related peptides as muscle relaxants in vertebrates may reflect an evolutionarily ancient role of CT-type neuropeptides that can be traced back to the common ancestor of deuterostomes.

## Materials and methods

### Animals

Live specimens of the starfish *A. rubens* (>3 cm in diameter) were collected at low tide from the Thanet coast, Kent, UK or were obtained from a fisherman based at Whitstable, Kent, UK. The starfish were maintained in a seawater aquarium at ~12°C and were fed weekly with mussels (*Mytilus edulis*). Smaller juvenile specimens of *A. rubens* (diameter 0.5–1.5 cm) were collected at the University of Gothenberg Sven Lovén Centre for Marine Infrastructure (Kristineberg, Sweden) and were fixed in Bouin's solution. Live specimens of the starfish *P. pectinifera* were collected at Cheongsapo of Busan, Korea, and maintained in a recirculating seawater system at 15°C until use; the starfish were fed with Manila clam (*Venerupis philippinarum or Ruditapes philippinarum*) every 3 days.

### Determination of the structure of ArCT

A transcript encoding an *A. rubens* calcitonin-type precursor in (ArCTP) was identified previously based on analysis of neural transcriptome sequence data (Semmens et al., 2016) and a cDNA encoding ArCTP has been cloned and sequenced (Mayorova et al., 2016). The predicted CT-type peptide derived from ArCTP is a 39-residue peptide with an amidated C-terminus and a disulfide bond between the two N-terminally located cysteine residues. To determine if this predicted structure of ArCT is correct, extracts of radial nerve cords from *A. rubens* were prepared as described previously (Lin et al., 2017b) and then were analyzed using mass spectrometry.

The pH of the radial nerve cord extract was adjusted to 7.8 using ammonium bicarbonate and then it was analyzed by LC-MS/MS to identify peptides in their native conformation. In addition, to reduce and alkylate disulfide bonds, samples of the extract were incubated with the reducing agent dithiothreitol (DTT) and the alkylating agent iodoacetamide. To produce shorter peptides with clearer fragmentation patterns, samples of the extract were digested with trypsin without reduction or alkylation. Reversed phase chromatography was used (Ultimate 3000 RSLCnano system—Thermo Fisher Scientific) to separate peptides prior to mass spectrometric analysis (Orbitrap Fusion—Thermo Fisher Scientific). Two columns were utilized, an Acclaim PepMap μ-precolumn cartridge 300 μm i.d. x 5 mm 5 μm 100 Å and an Acclaim PepMap RSLC 75 μm x 50 cm 2 μm 100 Å (Thermo Scientific). Mobile phase buffer A was 0.1% formic acid in water and mobile phase B was 0.1% formic acid in acetonitrile. Samples were loaded onto the μ-pre-column equilibrated in 2% aqueous acetonitrile containing 0.1% trifluoroacetic acid for 8 min at 10 μL min^−1^, after which peptides were eluted onto the analytical column at 300 nL min^−1^ by increasing the mobile phase B concentration from 4% B to 25% B over 39 min and then to 90% B over 3 min, followed by a 10 min re-equilibration at 4% B. Eluted peptides were converted to gas-phase ions by means of electrospray ionization and by high-energy collision dissociation (HCD) data-dependent fragmentation. All data were acquired in the Orbitrap mass analyser at a resolution of 30K. Survey scans of peptide precursors for HCD fragmentation were performed from 375 to 1500 m/z at 120K resolution (at 200 m/z), with automatic gain control (AGC) at 4 × 105 and isolation at 1.6 Th. The normalized collision energy for HCD was 33 with normal scan MS analysis and MS2 AGC set to 5.4 × 104 and the maximum injection time was 200 ms. Precursors with charge state 2–6 were selected and the instrument was run in top speed mode with 2 s cycles. The dynamic exclusion duration was set to 45 s with a 10 ppm tolerance around the selected precursor and its isotopes with monoisotopic precursor selection turned on.

Data analysis was performed by Proteome Discoverer 2.2 (Thermo Fisher Scientific) using ion mass tolerance of 0.050 Da and a parent ion tolerance of 10.0 ppm. Amidation of the C-terminus, oxidation of methionine and either dehydration or carbamidomethylation of cysteine were specified as variable modifications.

### Phylogenetic comparison of the sequences of ArCT and ArCTP with other calcitonin-related peptides/precursors

To compare the relationship of ArCTP with precursors of calcitonin-related peptides from other species, a multiple sequence alignment was generated using MUltiple Sequence Comparison by Log-Expectation (MUSCLE; https://www.ebi.ac.uk/Tools/msa/muscle/) and a phylogenetic tree was generated using the neighbor joining method with MEGA7 (http://www.megasoftware.net). In addition, the ArCT peptide sequence was aligned with related calcitonin-type peptides from other species using MAFFT (Multiple Alignment using Fast Fourier Transform; https://www.ebi.ac.uk/Tools/msa/mafft/). A figure showing the sequence alignment was produced using the BOXSHADE Server—EMBnet (version 3.21, https://embnet.vital-it.ch/software/BOX_form.html), with the fraction of sequences that must agree for shading set to 0.5. The full species names, accession numbers/citations are listed in Supplemental Table 1.

### Localization of ArCTP expression in *A. rubens* using mRNA *in situ* hybridization

Digoxigenin-labeled RNA antisense probes complementary to ArCTP transcripts and corresponding sense probes were synthesized, as reported previously (Mayorova et al., 2016). The methods employed for visualization of ArCTP expression in sections of the arms, central disk, or whole-body of *A. rubens* were the same as those reported previously for analysis of the expression of the relaxin-type precursor ArRGPP (Lin et al., 2017b), the ArGnRH and ArCRZ precursors (Tian et al., 2017), the pedal peptide-type precursor ArPPLNP1 (Lin et al., 2017a), and the luqin-type precursor ArLQP (Yañez-Guerra et al., 2018).

### Production and characterization of a rabbit antiserum to the C-terminal region of ArCT and immunohistochemical localization of ArCT in *A. rubens*

An antigen peptide, KY*NSPFGASGP-NH*_2_ (ArCT-ag), comprising an amidated nonapeptide corresponding to the C-terminal region of ArCT (in italics) was custom synthesized by Peptide Protein Research Ltd (Fareham, Hampshire, UK). An N-terminal lysine residue was incorporated to provide a reactive site for coupling to a carrier protein (thyroglobulin) and a tyrosine residue was incorporated so that the peptide could be used as a tracer for a ^125^I-based radioimmunoassay if required. A conjugate of the KYNSPFGASGP-NH_2_ peptide and thyroglobulin was prepared by using glutaraldehyde as a coupling reagent (Skowsky and Fisher, 1972). Antiserum production in a male rabbit was carried out by Charles River Ltd (Margate, UK; project code number 17582) using the same immunization and bleeding protocol reported previously for generation of an antiserum to ArPPLN1b (Lin et al., 2017a). To assess the presence and titer of antibodies to the antigen peptide, pre-immune serum, and antiserum from the final bleed were analyzed using an enzyme-linked immunosorbent assay (ELISA), employing use of protocols similar to those reported previously for an antiserum to the peptide ArPPLN1b (Lin et al., 2017a).

The methods employed for immunohistochemical localization of ArCT in *A. rubens* were the same as the methods reported previously for ArPPLN1b (Lin et al., 2017a), with the ArCT antiserum used at a dilution of 1:16,000. To test the specificity of the antiserum, the diluted antiserum [1:16000; in 5% goat serum/phosphate-buffered saline (PBS)] was incubated with ArCT-ag (200 μM) for 2 h at room temperature on a shaker. The pre-absorbed antiserum was then tested on starfish sections in parallel with tests using diluted antiserum.

### Analysis of the *in vitro* activity of ArCT on muscle preparations from *A. rubens*

Having confirmed the structure of ArCT using mass spectrometry, ArCT was custom synthesized (Peptide Protein Research Ltd, Fareham, Hampshire, UK). Informed by findings from analysis of the expression of ArCT in *A. rubens*, the effects of this peptide on *in vitro* preparations of neuromuscular organs from *A. rubens* were examined. ArCT was tested on three preparations, tube foot, apical muscle, and cardiac stomach, employing use of methods reported previously (Elphick et al., 1995; Melarange and Elphick, 2003; Lin et al., 2017a). The preparations were allowed to stabilize for 10 to 20 min in an organ bath containing aerated artificial seawater at ~11°C. To facilitate testing for relaxing effects of ArCT, tube foot and apical muscle preparations were induced to contract using 10 μM acetylcholine (ACh) and cardiac stomach preparations were induced to contract using 10 μM ACh or using artificial seawater (ASW) with 30 mM KCl added. Once a stable baseline contracted state was achieved, synthetic ArCT was added into the organ bath to sequentially achieve final concentrations in the range of 10^−9^-10^−6^ M or 10^−5^ M. These experiments were performed on at least three preparations from different animals. To quantify effects of ArCT, the change in muscle tone from a basal relaxed state prior to the fully contracted state following application of a muscle contractant (ACh or KCl) was designated as 100%. Relaxing effects of ArCT were then calculated as a percentage reversal of the 100% contracted state.

### Purification and identification of a muscle relaxant present in extracts of the starfish *P. pectinifera*

The presence of muscle relaxants in extracts of the starfish *P. pectinifera* has been reported recently (Kim et al., 2016) and one muscle relaxant named starfish myorelaxant peptide (SMP) has been identified as a pedal peptide/orcokinin-type neuropeptide (Kim et al., 2016). However, SMP is not the only muscle relaxant present in extracts of *P. pectinifera* because other myorelaxants with different chromatographic retention times to SMP are also detected. Here our objective was to determine the molecular identity of a myorelaxant from *P. pectinifera* that eluted between fractions 49 and 50 in the first purification step using a classical cation-exchange column chromatography (CM-52, 2.5 × 30 cm; Whatman, Maidstone, UK) (Kim et al., 2016). Bioactive fractions were pooled and then subjected to reversed phase (RP)-HPLC (Vydac 218TP510 Protein & Peptide C18, 9.2 × 250 mm; The Separation Group. Inc., Hesperia, CA, USA). Elution was performed with a linear gradient of 0–60% acetonitrile/0.1% trifluoroacetic acid (TFA) at a flow rate of 3.0 mL/min for 120 min, and fractions were collected every 2 min. Bioactive fractions were eluted between 34 and 36 min on RP-HPLC and these were subjected to further purification steps using an anion-exchange column (TSKgel DEAE-5PW, 7.5 × 75 mm; Tosho Corp., Minato-ku, Tokyo, Japan) with a linear gradient of 0–0.5 M sodium chloride in 20 mM Tris-HCl (pH 8.8) at a flow rate of 0.5 mL/min for 100 min. Material that was not retained on the anion-exchange column caused relaxation of the apical muscle from *P. pectinifera* and this material was pooled and subjected to further RP-HPLC (Hypersil-BDS C18, 2 × 125 mm; HP, Waldbronn, Germany) using a linear gradient of 20–40% acetonitrile/0.1% TFA at a flow rate 0.5 mL/min for 60 min. A bioactive peak eluted with 26% acetonitrile/0.1% TFA was then subjected to cation-exchange HPLC (TSKgel SP-5PW, 7.5 × 75 mm; Tosho Corp., Minato-ku, Tokyo, Japan) with a linear gradient of 0–1.0 M sodium chloride in 10 mM phosphate buffer (pH 6.0) at a flow rate 0.5 mL/min for 100 min. A single absorbance peak, which was eluted with 0.11 M sodium chloride, caused relaxation of the apical muscle from *P. pectinifera*. This peak was re-chromatographed by isocratic elution with 25% acetonitrile/0.1% TFA at a flow rate of 0.5 mL/min on the RP column used for the third purification step (Hypersil-BDS C18, 2 × 125 mm; HP, Waldbronn, Germany). The molecular mass of the purified peptide was determined by matrix-assisted laser desorption ionization (MALDI) time-of-flight (TOF) mass spectrometry (MS) (Voyager-DETM PRO spectrometer; Perseptive Biosystem, Framingham, MA, USA) using α-cyano-4-hydroxycinnamic acid (10 mg/ml in 50% acetonitrile/0.1% TFA) as a matrix. To determine the N-terminal amino acid sequence of the purified peptide, an aliquot of the purified peptide was dissolved in 10 μL of distilled water and then applied to a glass fiber disk after polybrene treatment. The glass fiber disk was dried off with nitrogen gas and then 5 μL of pyridylethylated reagent (0.16% 4-vinylpyridine, 0.08% tributylphosphine in 20% aqueous acetonitrile) was added to the glass fiber disk. The sequence analysis was carried out using an automated N-terminal amino acid gas-phase sequencer (PPSQ-33A; Shimadzu Corp. Nakagyo-ku, Kyoto, Japan). The purified peptide was identified as calcitonin-type peptide and named *Patiria pectinifera* calcitonin (PpCT).

### Cloning of a cDNA encoding the PpCT precursor (PpCTP) and analysis of its expression using RT-qPCR

A cDNA encoding the PpCT precursor protein was cloned using methods similar to those reported previously for cloning of the SMP precursor cDNA (Kim et al., 2016). Based on the amino acid sequence of PpCT, two degenerate primers were designed for 3′ RACE PCR, and then 5′ RACE PCRs were conducted with sequence-specific primers based on the sequencing result from the 3′ RACE product. The sequences of primers used for RACE PCR are listed in Supplemental Table 2. The cDNA sequence obtained (GenBank accession number MG832999) was translated into protein sequence using ExPASy (http://web.expasy.org/translate/) and SignalP 4.1 (http://www.cbs.dtu.dk/services/SignalP/) was used to predict the signal peptide of the translated protein sequence. To investigate the pattern of expression of PpCTP, five tissues from five specimens of *P. pectinfera* were analyzed using RT-qPCR, including the apical muscle, radial nerve cord, cardiac stomach, pyloric stomach and coelomic epithelium. Total RNA was extracted from the collected tissues using Hybrid-R (GeneAll, Seoul, Korea), and RNA quality was assessed by 1.0% agarose gel electrophoresis and then quantified spectrophotometrically using a Nano-Drop Lite (Thermo Fisher Scientific, Wilmington, MA, USA). cDNA was synthesized using the TOPscript cDNA synthesis kit with oligo dT (dT18; Enzynomics) according to manufacturer instructions. To validate differences in PpCT transcript expression in tissues, RT-qPCR was employed using a CFX Connect real-time PCR detection system (Bio-Rad Laboratories), as previously described (Kim et al., 2016). Briefly, amplification was performed in 20 μL volume reactions containing 10 μL of 2 × SYBR Green premix (TOPreal qPCR 29 PreMix; Enzynomics), 1 μL (10 pmol/μL) of each gene-specific forward and reverse primer, 1 μL of 10-fold-diluted cDNA template, and nuclease-free water added to make a final volume of 20 μL. Primer pairs used for amplifying PpCT-precursor, with elongation factor 1a (EF1α) (accession no.: GFOQ01387693) cDNA as a control for normalization (Sadritdinova et al., 2013), were PpCT qPCR-F and -R and EF1α qPCR-F and -R, respectively (Supplemental Table 2). The thermal-cycling profile was 95°C for 10 min, followed by 40 cycles at 95°C for 10 s, 60°C for 15 s, and 72°C for 15 s, with fluorescence recording at the end of each cycle. Melting-curve analysis was performed to ensure product specificity over the temperature range 60–90°C. Amplicons were analyzed on agarose gels to confirm product size. Based on the standard curves for both PpCT and EF1α, the relative expression levels of PpCT precursor transcripts in each tissue and at each time point were normalized against the level of EF1α using the comparative CT method (2^−ΔΔ*CT*^) (Livak and Schmittgen, 2001). Triplicate cDNA sample amplifications were performed independently, and comparison between tissues was carried out by one-way analysis of variance (ANOVA), followed by Duncan's multiple range test using GraphPad Prism software version 7.0 for Windows (GraphPad Software, San Diego, CA, USA). *P*-values less than 0.05 were considered statistically significant.

### Analysis of the *in vitro* activity of PpCT on apical muscle preparations from *P. pectinifera*

Based on the results of peptide structure analyses and precursor cDNA cloning, PpCT comprising a disulfide bond between the two cysteines and with C-terminal amidation was custom synthesized by Peptron Inc. (Daejeon, Korea). The synthetic peptide was re-purified to greater than 99% purity by RP-HPLC, and its structure was confirmed by MALDI-TOF MS. Apical muscle preparations dissected from *P. pectinifera* were used for *in vitro* bioassays to examine the effects of synthetic PpCT, employing previously reported methods (Kim et al., 2016). Briefly, the apical muscle was cut from the aboral body wall of an arm, and both ends of the muscle preparation were tied with cotton threads. The preparation was then suspended vertically in a 2 mL polypropylene chamber containing ASW with aeration, one end being connected to a silver hook on the bottom of the chamber and the other to a force displacement transducer (Type 45196A; NEC-Sanei Instrument Ltd., Tokyo, Japan). Output from the force displacement transducer was monitored by a recorder (WR7300; GRAPHTEC CORP., Yokohama, Japan) via an amplifier (AS1302; NEC-Sanei Instrument Ltd.), which recorded the mechanical responses of the device. Prior to testing, the muscle preparation was allowed to stabilize for about 90 min. The resting tension was then adjusted to 1.0 g. Apical muscle preparations were allowed to equilibrate for about 30 min in ASW, during which the ASW in the chamber was freshly replaced every 15 min. Contraction of apical muscle was induced by applying 1 μM acetylcholine (ACh). Then when a stable contracted state was achieved (~3 min), the muscle was treated with test samples to measure relaxation responses.

Five separate experiments were conducted to test the activity of synthetic PpCT using a concentration range of 10^−10^-10^−5^ M at 25°C. All data were expressed as means ± standard error of the mean (SEM). Dose-response curves were fitted with non-linear regression analysis and a sigmoidal curve of a four-parameter logistic equation with automatic outlier elimination using Prism software version 7.0 for Windows (GraphPad Software, San Diego, California, USA). EC_50_ value is represented with *p*EC_50_, the negative logarithm of half maximal effective concentration of the peptide, and E_max_ was expressed as the best-fit top value on a dose-response curve for peptide-induced relaxation.

## Results

### Confirmation of the structure of the *A. rubens* calcitonin-type neuropeptide ArCT

The predicted sequence of the ArCT precursor protein (ArCTP) based on analysis of assembled transcriptome sequence data (Semmens et al., 2016) has been confirmed by cDNA cloning and sequencing (Mayorova et al., 2016) (GenBank accession number KT601715). ArCTP is 114-residue precursor protein with a predicted 21-residue signal peptide and a 39-residue CT-like peptide sequence bounded by dibasic cleavage sites. Potential post-translational modifications of the CT-like peptide include formation of a disulfide bond between two cysteine residues located in the N-terminal region of the peptide and conversion of a C-terminal glycine residue to an amide group.

Analysis of extracts of *A. rubens* radial nerve cords using liquid chromatography and HCD data-dependent analysis mass spectrometry (LC-MS/MS) revealed the presence of a peptide under native conditions with a molecular mass [949.18 m/z (4+)] consistent with the predicted structure of ArCT: a 39-residue peptide with C-terminal amidation and a disulfide bond (NGESRGCSGFGGCGVLTIGHNAAMRMLAESNSPFGASGP-NH_2_, Supplementary Figure 1A). Furthermore, following reduction and alkylation a peptide was detected with a mass [978.20 m/z (4+)] that is consistent with the presence of a disulfide bond between the two cysteine residues in the native peptide (Supplementary Figure 1B). To further investigate the occurrence of a disulfide bond in ArCT, samples of radial nerve extract subjected to tryptic digestion without reduction and alkylation were analyzed. A good quality MS2 spectrum was obtained for the peptide GCSGFGGCGVLTIGHNAAMR with dehydrocysteine residues representing the disulfide bond (Supplementary Figure 1C). Evidence that the C-terminal proline of ArCT is amidated was provided by a −1 Da mass difference for the native peptide (Supplementary Figure 1A) and for the MLAESNSPFGASGP-NH_2_ peptide fragment (Supplementary Figure 1D) and the strong b ion series (as the amide group provides an additional basic group amenable to protonation).

### Comparison of the sequences of ArCTP and ArCT with other calcitonin-type precursors and peptides

**Figure 1:**
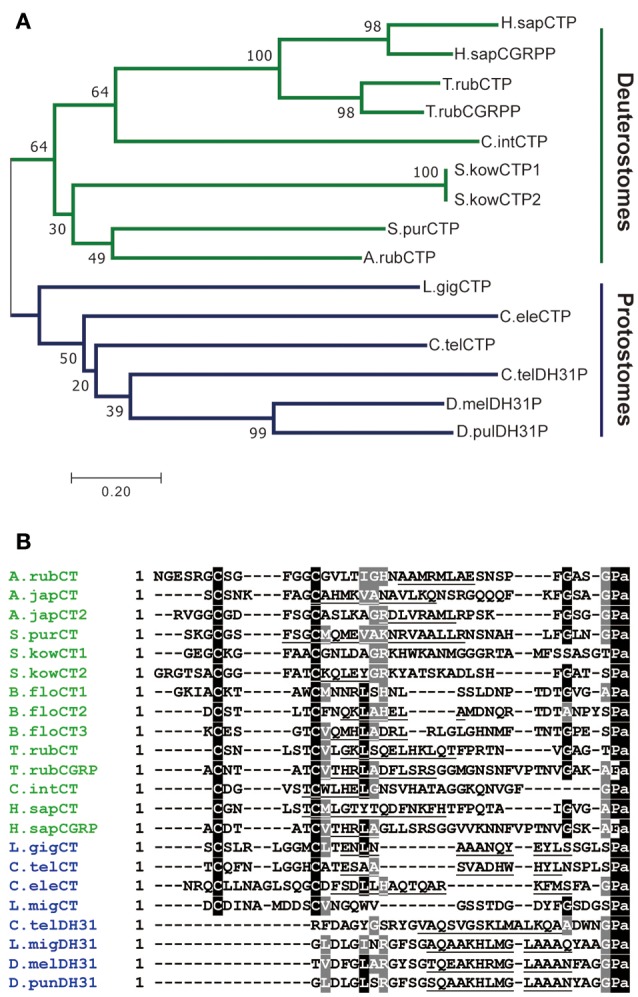
Comparison of ArCT and ArCTP with calcitonin-related peptides and precursors from other species. **(A)** Neighbor joining tree showing relationships of ArCTP with CT-type precursors from other species. Reflecting phylogenetic relationships, the tree comprises a deuterostomian clade (green) and a protostomian clade (blue) and ArCTP is positioned in a branch of the deuterostomian clade that includes CT-type precursors from other ambulacrarians—the sea urchin *Strongylocentrotus purpuratus* (phylum Echinodermata) and the acorn worm *Saccoglossus kowalevskii* (phylum Hemichordata). The scale bar indicates amino acid substitutions per site. **(B)** Comparison of the sequence of ArCT with CT-related peptides from other deuterostomes (green) and from protostomes (blue). A conserved feature of all of the peptides, except CGRP-type peptides, is an amidated C-terminal proline. With the exception of protostomian DH31-type peptides, all of the peptides have a pair of cysteine residues in the N-terminal region, which have been shown to form a disulfide bond in ArCT and in other CT-type peptides. Other residues that are conserved across many of the peptides are shown with white lettering highlighted in gray. Another conserved feature is a core region of the peptides (underlined) that is predicted to form an amphipathic α-helix. The full species names, accession numbers and/or citations for the sequences included in this figure are listed in Supplemental Table 1.

Comparison of ArCTP with other calcitonin-type precursors using the neighbor joining method yielded a tree reflective of animal phylogenetic relationships (Figure 1A), with distinct deuterostomian and protostomian clades. Furthermore, within the deuterostomian clade the tree structure reflected phylogenetic relationships, with a chordate clade that includes human CT and CGRP, pufferfish CT and CGRP and a urochordate CT and an ambulacrarian clade that includes ArCT and CT-type peptides from other echinoderms and hemichordates (Figure 1A). In the protostomian clade, there are two kinds of CT-type peptides, one type (CT) is like calcitonin in having an N-terminal disulfide bond, whilst the other type (DH31) lacks an N-terminal pair of cysteine residues (Figure 1A).

Comparison of the sequence of ArCT with other CT-related peptides revealed several conserved structural characteristics (Figure 1B). With the exception of vertebrate CGRPs, all of the peptides included in the alignment have a C-terminal Pro-NH_2_ motif, although the occurrence of C-terminal amidation has only been demonstrated biochemically for some of the peptides (including ArCT). The other conserved feature, except in DH31-type peptides, is an N-terminally located pair of cysteine residues, which form a disulfide bond in the mature peptides. As with C-terminal amidation, the occurrence of the N-terminal disulfide bond has been demonstrated in some (including ArCT) but not all of the peptides shown in Figure 1B. It is noteworthy that there appears to be a distinct difference in the number of residues separating the two cysteines in deuterostomian and protostomian CT-type peptides. Specifically, in deuterostomian CT-type peptides there are usually 4–5 residues (e.g., ArCT has 5), whereas in protostomian CT-type peptides there are 7–9 residues. Another feature that is conserved amongst many of the CT-type peptides aligned in Figure 1B is a glycine residue located at the fifth position from the C-terminal amide. Furthermore, analysis of secondary structure predicts the presence of an amphipathic α-helix in the central core of the majority of the peptides aligned in Figure 1B (including ArCT).

### Localization of ArCTP transcript in *A. rubens* using mRNA *in situ* hybridization

**Figure 2:**
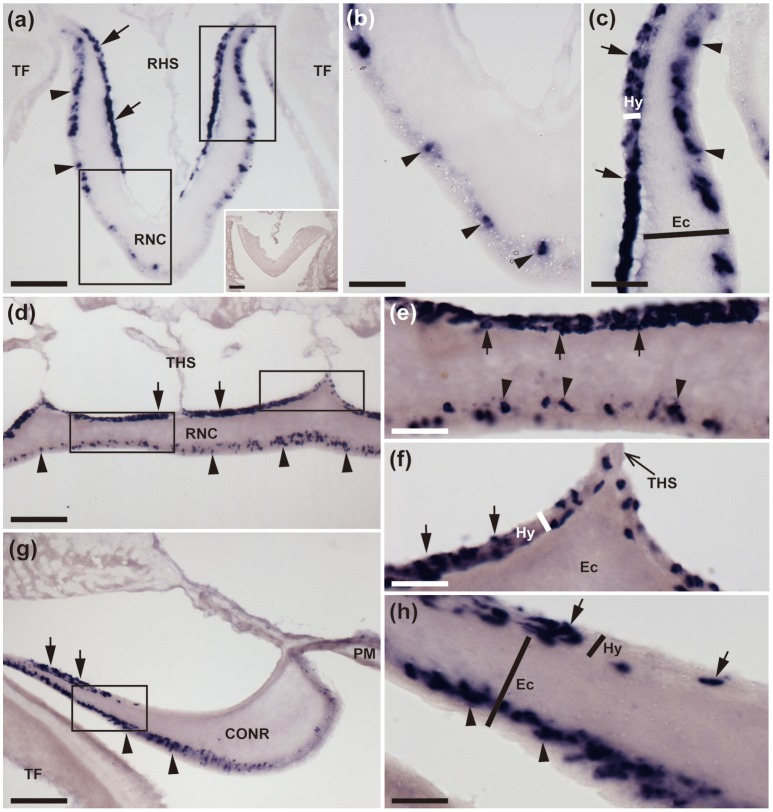
Localization of ArCTP mRNA in the radial nerve cords and circumoral nerve ring of *A. rubens* using *in situ* hybridization. **(a)** Transverse section of a radial nerve cord that was incubated with ArCTP antisense probes showing stained cells in both the ectoneural (arrowheads) and hyponeural (arrow) regions. In the ectoneural region stained cells are concentrated laterally in the sub-cuticular epithelium, whereas in the hyponeural region stained cells are more uniformly distributed. Higher magnification images of the boxed regions are shown in panels **(b,c)**. The inset shows an absence of stained cells in a transverse section of radial nerve cord incubated with ArCTP sense probes, demonstrating the specificity of staining observed with ArCTP antisense probes. **(b)** High magnification image of the apex of the V-shaped radial nerve cord, where stained cells are sparsely distributed in the sub-cuticular epithelial layer of the ectoneural region (arrowheads). **(c)** High magnification image of the lateral region of the radial nerve cord showing stained cells in both the ectoneural region (arrowheads) and the hyponeural region (arrows). **(d)** Longitudinal parasagittal section of a radial nerve cord showing that stained cells are evenly distributed along its length in the ectoneural region (arrowheads). In the hyponeural region stained cells are in segmental clusters (arrows), with each segment of the hyponeural region bounded by transverse hemal strands. Higher magnification images of the boxed regions are shown in **(e,f)**. **(e)** Stained cells in the ectoneural (arrowheads) and hyponeural (arrows) regions of the radial nerve cord. **(f)** Junction of the hyponeural region of the radial nerve cord and a transverse hemal strand, showing that the density of stained cells is lower in this region than in the adjacent region (arrows). **(g)** Transverse section of the central disk region showing stained cells in both the hyponeural (arrows) and ectoneural (arrowheads) regions of the circumoral nerve ring. As in the radial nerve cords, stained cells are concentrated laterally in the ectoneural region. A high magnification image of the boxed region is displayed in panel **(h)**, which shows stained cells in the ectoneural (arrowheads) and hyponeural (arrows) regions of the circumoral nerve ring. CONR, Circumoral nerve ring; Ec, Ectoneural region of radial nerve cord; Hy, Hyponeural region of radial nerve cord; PM, Peristomial membrane; RHS, Radial hemal strand; RNC, Radial nerve cord; TF, Tube foot; THS, Transverse hemal strand. Scale bar: 100 μm in (**a,d,g)**; 50 μm in (**A** inset); 25 μm in **(b,c,e,f,h)**.

**Figure 3:**
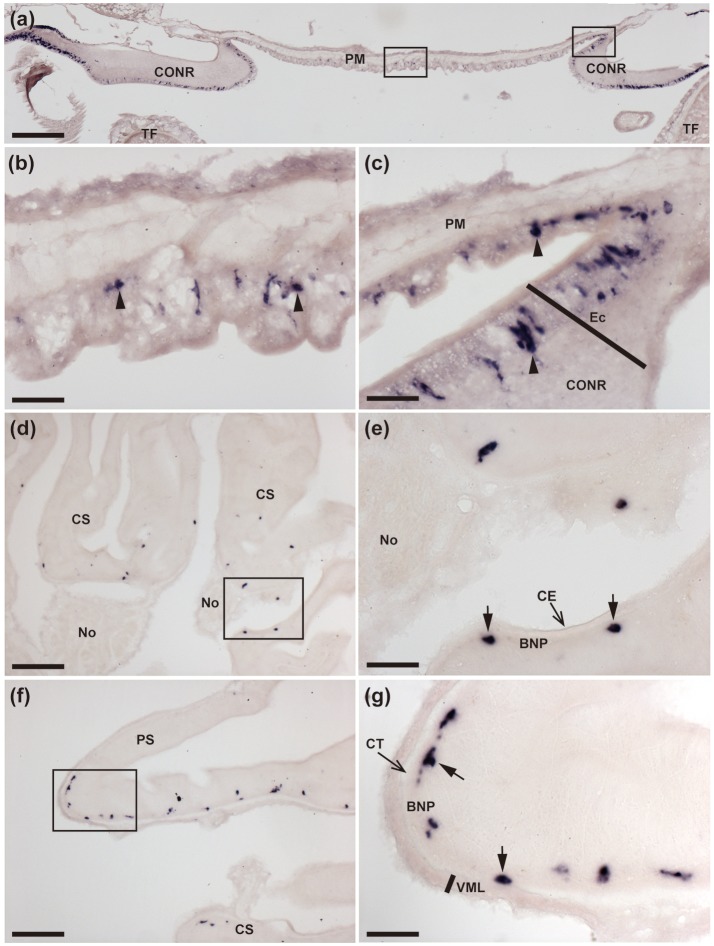
Localization of ArCTP mRNA in the peristomial membrane and stomach of *A. rubens* using *in situ* hybridization. **(a)** Transverse section through the central disk region showing staining in the peristomial membrane and in the circumoral nerve ring that surrounds the peristomial membrane. High magnification images of the boxed regions are shown in **(b,c)**. **(b)** Stained cells (arrowheads) in the external epithelium of the peristomial membrane. **(c)** Junction between the peristomial membrane and the circumoral nerve ring, with stained cells (arrowheads) in the external epithelium of the peristomial membrane and in the ectoneural epithelial layer of the circumoral nerve ring. **(d)** Stained cells in a section of cardiac stomach at the junction between an arm and the central disk, where the cardiac stomach is attached via nodules to extrinsic retractor strands. The boxed region is shown at higher magnification in **(e)**, where stained cells (arrows) can be seen to be located in the basi-epithelial nerve plexus layer. **(f)** Transverse section through the central disk region showing stained cells in both the cardiac stomach and pyloric stomach. The boxed region in **(f)** is shown at a higher magnification in **(g)**, where the stained cells (arrows) are located in the basi-epithelial nerve plexus layer. BNP, Basi-epithelial nerve plexus layer; CE, Coelomic epithelium; CONR, Circumoral nerve ring; CS, Cardiac stomach; CT, Collagenous tissue; Ec, Ectoneural region of radial nerve cord; No, Nodule; PM, Peristomial membrane; PS, Pyloric stomach; TF, Tube foot; VML, Visceral muscle layer. Scale bar: 250 μm in **(a)**; 100 μm in **(d,f)**; 25 μm in (**b,c,e,g**).

**Figure 4:**
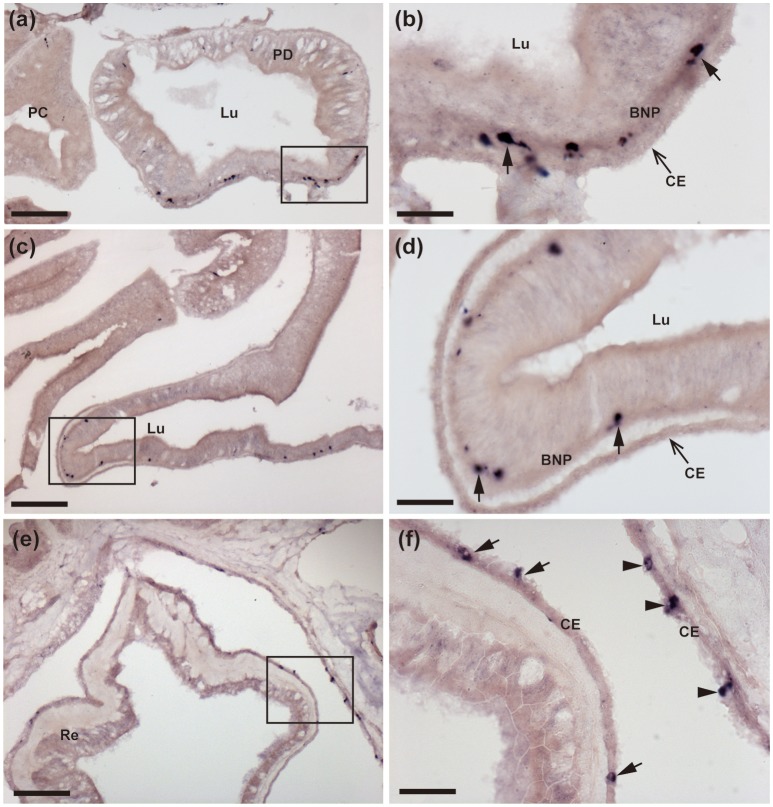
Localization of ArCTP mRNA in the pyloric ducts, pyloric caeca and rectal caeca of *A. rubens* using *in situ* hybridization. **(a,b)** Transverse section of a pyloric duct showing stained cells located on the oral side. The boxed region in **(a)** is shown at higher magnification in **(b)**, where stained cells (arrows) can be seen to be located in the basi-epithelial nerve plexus layer. **(c,d)** Transverse section of an arm showing stained cells in a pyloric caecum diverticulum. The boxed region in **(C)** is shown at higher magnification in **(d)**, where the stained cells (arrows) can be seen to be located in the basi-epithelial nerve plexus layer. **(e,f)** Transverse section of the central disk region showing stained cells in the rectum. The boxed region in (e) is shown at higher magnification in **(f)**, where stained cells can be seen to be located in the coelomic epithelial lining of the rectum (arrows) and the body wall (arrowheads). BNP, basi-epithelial nerve plexus layer; CE, Coelomic epithelium; PC, Pyloric caecum; PD, Pyloric duct; Lu, Lumen. Scale bar: 100 μm in **(a,c,e)**, 25 μm in **(b,d,f)**.

**Figure 5:**
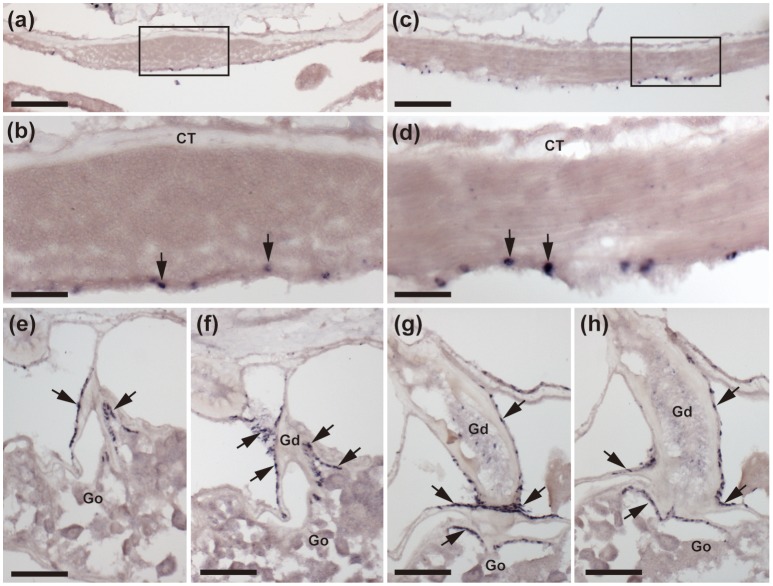
Localization of ArCTP mRNA in body wall-associated tissues and organs in *A. rubens* using *in situ* hybridization. **(a)** Transverse section of an arm showing staining in the apical muscle. The boxed region is shown at higher magnification in **(b)**, where stained cells (arrows) are located in the coelomic epithelial lining of the apical muscle. **(c)** Sagittal section of an arm showing that staining is present along the length of the apical muscle. The boxed region is shown at higher magnification in **(d)**, where stained cells (arrows) are located in the coelomic epithelial lining of the apical muscle. **(e–h)** A series of transverse sections of an arm showing staining in the gonoduct that links the gonad to the gonopore in the body wall. Stained cells (arrows) are present in the coelomic epithelium of the gonoduct and at the junction between the gonoduct and the gonad. CT, Connective tissue; Go, Gonad; Gd, Gonoduct. Scale bar: 100 μm in **(a,c)**; 25 μm in **(b,d)**; 50 μm in (**e,f,g,h)**.

In previous papers we have presented overviews of the anatomy of *A. rubens* to facilitate interpretation of the patterns of neuropeptide expression revealed by mRNA *in situ* hybridization and immunohistochemistry (Lin et al., 2017a; Tian et al., 2017; Yañez-Guerra et al., 2018). Analysis of the expression of ArCTP in *A. rubens* revealed a widespread pattern of expression, including the nervous system (Figure 2), digestive system (Figures 3, 4), and body wall-associated structures (Figure 5).

#### Nervous system

The starfish nervous system comprises radial nerve cords located in each of the five arms and linked by a circumoral nerve ring located in the central disk region. The radial nerve cords and circumoral nerve ring comprise two distinct regions—the ectoneural region, which contains sensory neurons and interneurons, and the hyponeural region, which contains motor neurons (Pentreath and Cobb, 1972; Mashanov et al., 2016). Cells expressing ArCTP transcripts were revealed in both the ectoneural and hyponeural regions of the radial nerve cords (Figure 2a). Absence of stained cells in sections incubated with ArCTP sense probes (Figure 2a inset) confirmed the specificity of staining observed with ArCTP antisense probes. Likewise, the specificity of staining observed in other regions of the body with ArCTP antisense probes (see below) was confirmed in tests using ArCTP sense probes, where no staining was observed (data not shown). Transverse sections of radial nerve cords show that in the ectoneural region the stained cells are concentrated laterally (Figures 2a,c) with the apex of the V-shaped nerve cord containing a sparser population of stained cells (Figures 2a,b), whereas in the hyponeural region stained cells are distributed uniformly (Figures 2a,c). In longitudinal sections of radial nerve cords, stained cells can be seen to be distributed along the length of ectoneural epithelium (Figures 2d,e), whereas in the hyponeural region there is segmental clustering of stained cells (Figures 2d–f). The pattern of ArCTP transcript expression in the circumoral nerve ring is generally consistent with that in the radial nerve cords, with stained cells concentrated laterally in both the ectoneural and hyponeural regions (Figures 2g,h).

#### Digestive system and body wall-associated tissues

The digestive system of *A. rubens* comprises several distinct regions. The mouth, which is located on the underside of the central disk, is surrounded by a peristomial membrane that is connected to a short esophagus. The bulk of the digestive system in the central disk comprises the cardiac stomach, which is everted through the mouth during feeding. Aboral to the cardiac stomach is the much smaller pyloric stomach, which is linked by pyloric ducts to the paired pyloric caeca located in each arm. Aboral to the pyloric stomach is a short rectum and associated rectal caeca, with fecal material voided from a tiny anus on the aboral surface of the central disk (Anderson, 1960; Jangoux and van Impe, 1977).

ArCTP transcripts were revealed in several regions of the digestive system. In the peristomial membrane, stained cells were observed in the external epithelium and at the margins of the peristomial membrane these stained cells are in close proximity to stained cells in the adjacent ectoneural epithelium of the circumoral nerve ring (Figures 3a–c). In both the cardiac stomach and pyloric stomach stained cells were observed closely associated with the basi-epithelial nerve plexus that is located at the boundary between the mucosa and a layer of collagenous tissue (Figures 3d–g). In the pyloric ducts, stained cells were concentrated on the oral side and, as in the cardiac stomach and pyloric stomach, located in close association with the basi-epithelial nerve plexus (Figures 4a,b). Stained cells were also revealed in close association with the basi-epithelial nerve plexus in diverticulae of the pyloric caeca (Figures 4c,d) and in the coelomic epithelial layer of the rectum (Figures 4e,f).

Stained cells were observed in the coelomic epithelial lining of the body wall, including cells located in the coelomic epithelial layer of the apical muscle (Figures 5a–d). ArCTP transcript expression was also observed in cells located in the coelomic epithelial layer of the gonoduct and at the junction between the gonoduct and the gonad (Figures 5e–h).

### Characterization of a rabbit antiserum to ArCT

ELISA analysis of antiserum from the final bleed revealed that the antigen peptide (1 × 10^−10^ mol per well) could be detected with antiserum dilutions as low as 1:32,000 (Supplementary Figure 2A). Furthermore, when tested at an antiserum dilution of 1:16,000, the antigen peptide could still be detected with as little as 0.1 pmol per well (Supplementary Figure 2B). Collectively, these data indicate that the antiserum contains a high titer of antibodies to the ArCT antigen peptide. The rabbit antiserum to ArCT has been assigned the RRID:AB_2721239.

### Localization of ArCT in *A. rubens* using immunohistochemistry

**Figure 6:**
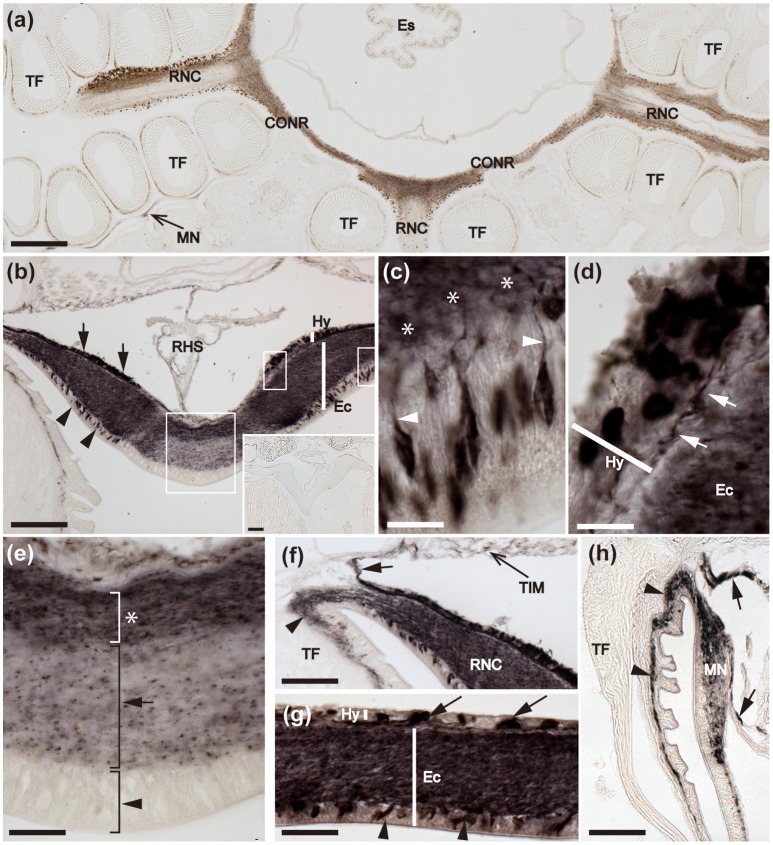
Immunohistochemical localization of ArCT in the radial nerve cords, circumoral nerve ring and marginal nerve of *A. rubens*. **(a)** Horizontal section of a juvenile specimen showing the presence of immunostaining in the circumoral nerve ring, radial nerve cords and marginal nerves. **(b)** Immunostained transverse section of the V-shaped radial nerve cord showing the presence of ArCT-immunoreactive cells in both the ectoneural (arrowheads) and hyponeural (arrows) regions. In the ectoneural epithelial layer the stained cells are concentrated laterally and no stained cells are present in apical region. The inset shows an absence of staining in sections of radial nerve cord incubated with antiserum that was pre-absorbed with the ArCT peptide antigen, demonstrating the specificity of immunostaining observed in sections incubated with the antiserum. The boxed regions are displayed at high magnification in **(c–e)**. **(c)** Immunostained bipolar cells in the ectoneural epithelium with stained processes (arrowheads) projecting into the underlying stained neuropile (*). **(d)** Immunostained monopolar cells in the hyponeural region with stained processes (arrows), that run parallel to the collagenous tissue layer between the hyponeural and ectoneural regions. **(e)** Apical region of a radial nerve cord showing absence of stained cells in the ectoneural epithelium (arrowhead), with sparse staining of fibers in the underlying neuropile (arrow). Denser staining is present in the inner region of ectoneural neuropile (asterisk). **(f)** Immunostaining at the junction between a radial nerve cord and an adjacent tube foot. Immunostained processes (arrowhead) project from the ectoneural neuropile into the basi-epithelial nerve plexus of the tube foot. Immunostained processes (arrow) derived from the hyponeural region project around the margin of the peri-hemal canal to innervate the transverse infra-ambulacral muscle. **(g)** Immunostaining in a longitudinal section of the circumoral nerve ring showing immunostained cells in the ectoneural epithelium (arrowheads) and in the hyponeural region (arrows). Intense staining is present throughout the ectoneural neuropile. **(h)** Immunostaining in the marginal nerve and in the basi-epithelial nerve plexus (arrowheads) of an adjacent tube foot. Immunostained processes of the lateral motor nerve can also be seen here (arrows). CONR, Circumoral nerve ring; Ec, Ectoneural region of radial nerve cord; Es, Esophagus; Hy, Hyponeural region of radial nerve cord; MN, Marginal nerve; OS, Ossicle; RHS, Radial hemal strand; RNC, Radial nerve cord; TF, Tube foot; TIM, Transverse infra-ambulacral muscle. Scale bar: 250 μm in **(a)**; 100 μm in **(b)**; 50 μm in **(b)** inset; 10 μm in **(c–e)**; 50 μm in **(f,h)**; 25 μm in **(g)**.

Immunohistochemical analysis of ArCT expression in *A. rubens* revealed a widespread pattern of immunostaining that was consistent with the distribution of cells expressing the ArCTP transcript. This is illustrated in a low magnification image of a horizontal section of a juvenile starfish (Figure 6a), with immunostaining seen in the radial nerve cords, circumoral nerve ring, marginal nerves, and the basi-epithelial plexus of tube feet. The specificity of immunostaining in the radial nerve cords and in other regions of the starfish body was confirmed by pre-absorption tests where immunostaining (exemplified with radial nerve cord in Figure 6b) was abolished by incubation of antiserum with the antigen peptide (Figure 6b inset).

#### Nervous system

Detailed analysis of immunostained transverse sections of radial nerve cords reveals stained cells in the lateral regions of the ectoneural epithelium and stained processes in the underlying neuropile (Figures 6b,c). High magnification images reveal the bipolar shape of these cells, with a process projecting into the neuropile (Figure 6c). Immunostained monopolar cells in the hyponeural region of the radial nerve cord are roundish in shape with a process projecting into an underlying nerve plexus (Figures 6b,d). In the apical region of the radial nerve cord where there is an absence of stained cells in the ectoneural epithelium, the underlying neuropile exhibited weaker immunostaining than in other regions of the ectoneural neuropile (Figure 6e). Immunostained processes derived from the ectoneural neuropile can be seen to project into the basi-epithelial plexus of adjacent tube feet, whilst immunostained processes derived from the hyponeural region can be seen to project to the infra-ambulacral muscle (Figure 6f). In the circumoral nerve ring the pattern of immunostaining observed is similar to that in the radial nerve cords, with stained cells and processes in both the ectoneural and hyponeural regions (Figures 6a,g). Immunostaining is also present in the marginal nerves, which are longitudinal thickenings of the sub-epithelial nerve plexus located lateral to the outer row of tube feet (Figures 6a,h). Furthermore, internal to the marginal nerves are immunostained processes associated with the lateral motor nerves (Figure 6h).

#### Tube feet

**Figure 7:**
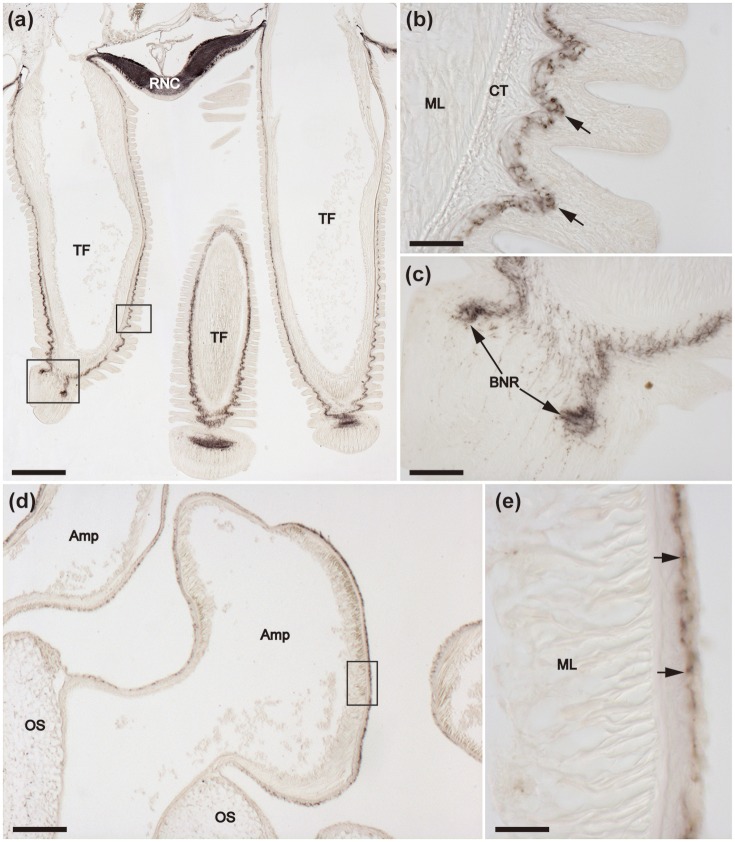
Immunohistochemical localization of ArCT in tube feet of *A. rubens*. **(a)** Tranverse section of an arm showing the presence of immunostaining in the radial nerve cord and adjacent tube feet. Immunostaining is present in the sub-epithelial nerve plexus along the length of the stem of each tube foot and extends into the basal nerve ring in the tube foot disk. The boxed areas are shown at high magnification in **(b,c)**. **(b)** Immunostaining in the sub-epithelial nerve plexus in the stem of a tube foot. **(c)** Immunostaining in the basal nerve ring of a tube foot disk. **(d)** Immunostaining in the ampulla of a tube foot. The boxed region is shown at higher magnification in **(e)**, which shows that the immunostaining is present in fibers located beneath the coelomic lining of the ampulla. Amp, Ampulla; BNR, Basal nerve ring; CT, Collagenous tissue; ML, Muscle layer; OS, Ossicle; RNC, Radial nerve cord; TF, Tube foot. Scale bar: 250 μm in **(a)**; 200 μm in **(d)**; 50 μm in **(b,c,e)**.

The presence of immunostaining in the basi-epithelial nerve plexus of the tube feet can be seen in the low magnification image of a horizontal section of a juvenile specimen of *A. rubens* shown in Figure 6a. The overall distribution of immunostaining in tube feet can be seen in transverse sections of arms, where tube feet are sectioned longitudinally (Figure 7a). Immunostained fibers in the basi-epithelial nerve plexus of the tube feet are contiguous with the ectoneural neuropile of the adjacent radial nerve cord (Figures 6f, 7a), and extend along the length of the tube foot stem (Figures 7a,b) to the disk region, where stained fibers are associated with the basal nerve ring (Figures 7a,c). Immunostained processes are also present in the nerve plexus underlying the coelomic epithelium of the tube foot ampullae (Figures 7d,e).

#### Digestive system

**Figure 8:**
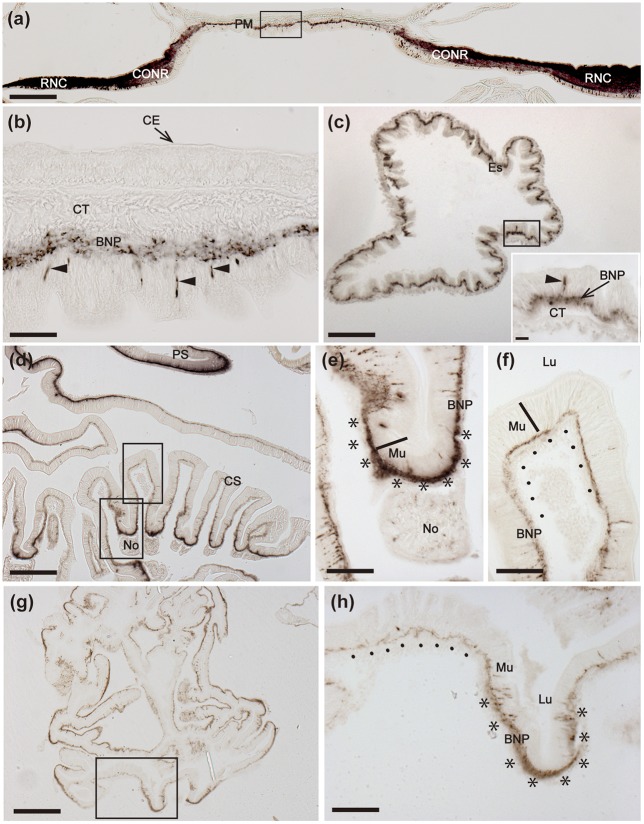
Immunohistochemical localization of ArCT in the peristomial membrane, esophagus and cardiac stomach of *A. rubens*. **(a)** Transverse section of the central disk region showing immunostaining in the radial nerve cords, circumoral nerve ring and peristomial membrane. The boxed region is displayed at higher magnification in **(b)**, which shows immunostained cells (arrowheads) in the external epithelium of the peristomial membrane and immunostained fibers in the underlying basi-epithelial nerve plexus. **(c)** Horizontal section of the central disk region showing immunostained fibers in the esophagus. The inset shows the esophagus at high magnification, with a stained cell in the mucosal layer (arrowhead) and staining in the underlying basi-epithelial nerve plexus. **(d)** Transverse section of the central disk region showing both the cardiac stomach and pyloric stomach. Note that there is variation in the density of immunostaining in the basi-epithelial nerve plexus in the folds of the cardiac stomach wall, as exemplified by the two boxed areas that are shown at high magnification in **(e,f)**. **(e)** Region of the cardiac stomach wall where it is attached to a nodule, which links the cardiac stomach to extrinsic retractor strands; here stained bipolar cells can be seen in the mucosal wall and the prominent basi-epithelial nerve plexus is intensely stained (^********^). Immunostained fibers can also be seen in the nodule. **(f)** Region of the cardiac stomach where stained cells are sparsely distributed and the underlying basi-epithelial nerve plexus is less intensely stained (……….) than in the adjacent region shown in **(e)**. **(g)** Horizontal section of the central disk region of a juvenile starfish showing immunostaining in the cardiac stomach. The boxed region is displayed at higher magnification in **(h)**, which shows variation in the intensity of immunostaining, with a folded region containing stained cells and a thickened and intensely stained basi-epithelial nerve plexus (^********^) and an adjacent region without stained cells and a less prominently stained basi-epithelial nerve plexus (…….). BNP, Basi-epithelial nerve plexus; CS, Cardiac stomach; CONR, Circumoral nerve ring; CE, Coelomic epithelium; CT, Collagenous tissue; Es, Esophagus; Lu, Lumen; Mu, Mucosa; No, Nodule; PM, Peristomial membrane; PS, Pyloric stomach; RNC, Radial nerve cord. Scale bar: 250 μm in **(a)**; 25 μm in **(b,c)** inset; 200 μm in **(d,g)**; 100 μm in **(c)**; 50 μm in **(e,f,h)**.

Immunostained cells are present in the external epithelial layer of the peristomial membrane, with stained processes projecting into the underlying basi-epithelial nerve plexus (Figure 8a,b). The staining in the basi-epithelial nerve plexus of the peristomial membrane is contiguous with the immunostained ectoneural region of the circumoral nerve ring, which is located at the outer margin of the peristomial membrane (Figure 8a). Immunostained fibers are also present in the basi-epithelial nerve plexus of the esophagus, which is located between the peristomial membrane and the cardiac stomach (Figure 8c). Extensive immunostaining is present in the cardiac stomach (Figures 8d–h), with immunostained bipolar cells located in the mucosal layer and immunostained fibers in the underlying basi-epithelial nerve plexus (Figures 8e,f,h). It is noteworthy, however, that the distribution of immunostaining in the folded wall of the cardiac stomach is not homogeneous; thus, some regions contain more stained cells and have a thicker/more intensely stained underlying basi-epithelial nerve plexus (Figures 8e,h), whilst adjacent regions contain fewer stained cells and a thinner/less intensely stained underlying basi-epithelial nerve plexus (Figures 8f,h).

**Figure 9:**
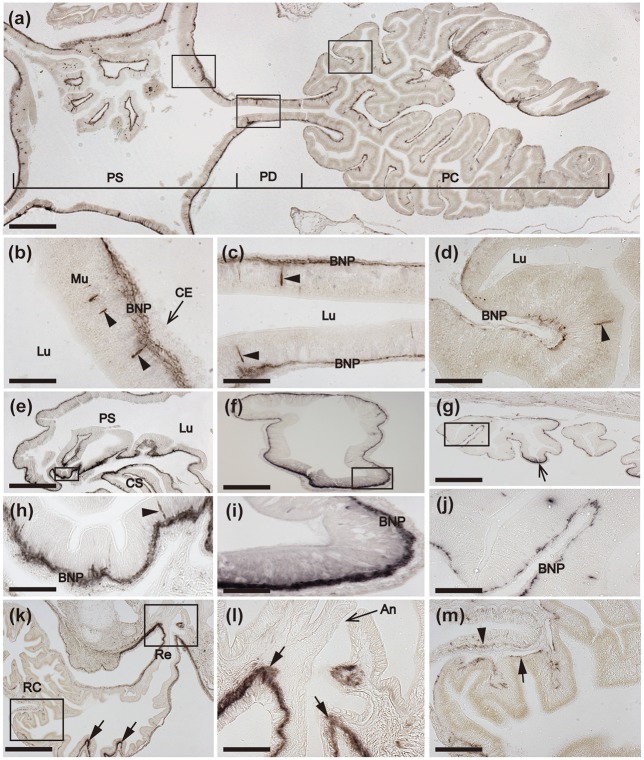
Immunohistochemical localization of ArCT in the pyloric stomach, pyloric ducts, pyloric caeca, rectum, and rectal caeca of *A. rubens*. **(a)** Horizontal section of a juvenile specimen showing the presence of immunostaining in the pyloric stomach, pyloric ducts, and pyloric caeca. High magnification images of the boxed regions are shown in **(b–d)**. **(b)** Immunostained cells (arrowheads) in the mucosa of the pyloric stomach with associated immunostained fibers in the underlying basi-epithelial nerve plexus. **(c)** Horizontal section of a pyloric duct showing immunostained cells (arrowheads) in the mucosa with associated immunostained fibers in the underlying basi-epithelial nerve plexus. **(d)** Immunostained cells (arrowheads) in the mucosa and immunostaining in the basi-epithelial nerve plexus of a pyloric caecum diverticulum. **(e)** Transverse section of the central disk region showing the distribution of immunostaining in the pyloric stomach. A high magnification image of the boxed region is displayed in **(h)**, which shows an immunostained cell (arrowhead) in the mucosa and immunostained fibers in the basi-epithelial nerve plexus. **(f,g)** Transverse sections of a pyloric duct **(f)** and a pyloric caecum **(g)** showing immunostained fibers in the basi-epithelial nerve plexus; note that the staining in the pyloric duct is more prominent on the oral (lower) side, as shown at higher magnification in **(i)**. **(j)** High magnification image of the boxed region in **(g)**, showing immunostaining in the basi-epithelial nerve plexus of a pyloric caecum diverticulum. **(k)** Transverse section of the central disk region showing immunostaining in the rectal caeca and rectum. High magnification images of the boxed regions are displayed in **(l,m)**. Immunostaining can also be seen in the inner region of the rectum (arrows) that is linked to the pyloric stomach. **(l)**. Junction between the rectum and the anal opening in the aboral body wall of the central disk; note the instense staining in the nerve plexus (arrows) beneath the coelomic epithelium of the rectum. **(m)** Immunostaining in both the visceral muscle layer (arrowhead) and sub-mucosal basi-epithelial nerve plexus (arrow) of a rectal cecum. An, Anus; BNP, Basi-epithelial nerve plexus; CS, Cardiac stomach; Ce, Coelomic epithelium; Lu, Lumen; Mu, Mucosa; PC, Pyloric caecum; PD, Pyloric duct; PS, Pyloric stomach; Re, Rectum; RC, Rectal caecum. Scale bar: 250 μm in **(a)**; 150 μm in **(e,g,k)**; 100 μm in **(f)**; 40 μm in **(j,l,m)**; 25 μm in **(b,c,d,h)**; 20 μm in **(i)**.

Immunostained cells and their processes in the underlying basi-epithelial nerve plexus are also present in the pyloric stomach (Figures 8d, 9a,b,e,h), which is located aboral to the cardiac stomach and is linked by pyloric ducts to the paired pyloric caeca located in each arm (Figure 9a). In the pyloric ducts, immunostained cells and their processes in the underlying basi-epithelial nerve plexus are illustrated in both horizontal (Figure 9c) and transverse (Figure 9f) sections, with the latter showing that the staining in the basi-epithelial nerve plexus is thicker/more intensely stained on the oral side of the duct than on the aboral side. In the pyloric caeca, staining is present throughout the basi-epithelial layer but it is thicker/more intense in the ducts of the pyloric caeca than in the diverticulae (Figures 9d,g,j).

The pyloric stomach is linked to the aborally-located anus by a rectum, which has associated rectal caeca. An intensely stained nerve plexus is present in the rectum, which is located beneath the coelomic epithelium and is contiguous with the sub-epithelial plexus of the adjacent body wall coelomic lining (Figures 9k,l). Less prominent immunostaining is present in nerve plexi of the rectal caecae (Figures 9k,m).

#### Body-wall associated tissues/organs

**Figure 10:**
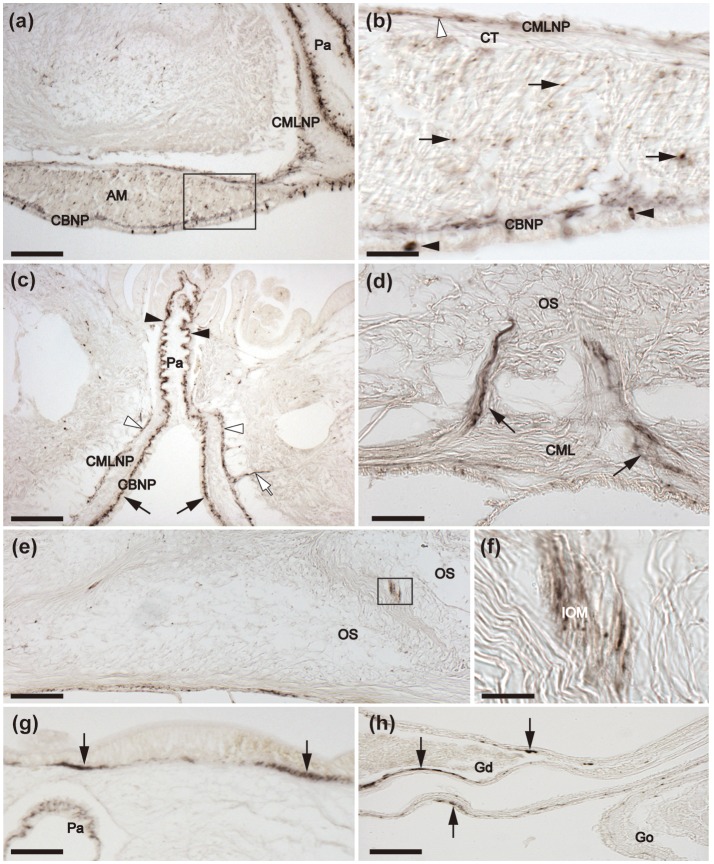
Immunohistochemical localization of ArCT in body wall-associated tissues/organs in *A. rubens*. **(a)** Tranverse section of an arm showing immunostaining in the apical muscle and an adjacent papula. A high magnification image of the boxed region is shown in **(b)**. **(b)** Immunostained cells (black arrowheads) are present in the coelomic lining of the apical muscle and profiles of immunostained fibers (arrows) are amongst the longitudinally orientated muscle fibers of the apical muscle. Immunostained fibers can also be seen here in the circular muscle layer of the body wall (white arrowhead). **(c)** Transverse section of an arm showing the presence of immunostaining in the nerve plexus beneath the coelomic epithelial lining of a papula (arrowheads), which is contiguous with the basi-epithelial nerve plexus of the epithelium lining the main coelomic cavity of the arm (arrows). Immunostaining can also be seen here in association the circular muscle layer (white arrowheads) and an immunostained process derived from this layer can be seen projecting into the body wall (white arrow). **(d)** Immunostained nerve fibers (arrows) associated with strands of muscle that are attached to body wall ossicles and are derived from the circular muscle layer of the body wall **(e)** Transverse section of the aboral body wall of an arm, showing immunostained fibers associated with interossicular muscles, with the boxed region shown at higher magnification in **(f)**. **(g)** Immunostaining in the sub-epithelial nerve plexus (arrows) of the aboral body wall external epithelium. **(h)** Transverse section of an arm showing immunostaining (arrows) in the gonoduct that connects the gonad to the gonopore. AM, apical muscle; CBNP, Coelomic basi-epithelial nerve plexus; CML, circular muscle layer; CMLNP, Circular muscle layer nerve plexus; CT, collagenous tissue; Go, Gonad; Gd, Gonoduct; IOM, Inter-ossicular muscle; OS, Ossicle; Pa, Papula; PC, Pyloric caecum; Scale bar: 200 μm in **(a,c,e)**; 100 μm in **(g,h)**; 25 μm in **(b,d)**; 10 μm in **(f)**.

Immunostained cells are present in the coelomic epithelium that lines the body wall, as illustrated in Figures 10a,b, which shows stained cells in the coelomic epithelial layer of the apical muscle. Immunostained processes derived from these cells ramify amongst the longitudinally orientated muscle fibers of the apical muscle (Figure 10b). Immunostained processes can also be seen in the circularly-orientated muscle layer of the body wall, which is separated from the longitudinally orientated muscle by a thin layer of collagenous tissue (Figures 10b,c). The immunostained nerve plexus associated with the longitudinally orientated muscle layer of the body wall also extends along the length of the walls of papulae, finger-shaped appendages that enable gas exchange between the coelomic fluid and the external seawater (Figures 10a,c). Immunostained nerve fibers are also associated with strands of muscle derived from the circular muscle layer that are attached to ossicles in the aboral body wall (Figure 10d) and with interossicular muscles (Figures 10e,f). Immunostaining is also present in the sub-epithelial nerve plexus of the external body wall epithelium (Figure 10g) and in the paired gonoducts of each arm, which link the gonads to gonopores (Figure 10h).

### ArCT causes dose-dependent relaxation of tube foot and apical muscle preparations from *A. rubens*

**Figure 11:**
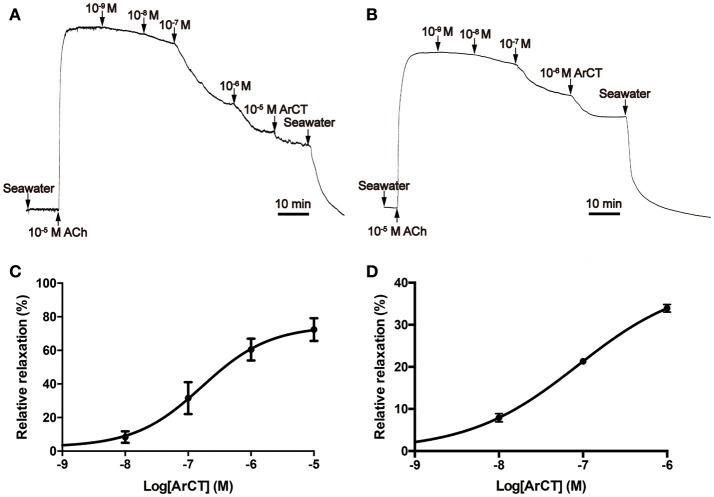
ArCT causes dose-dependent relaxation of tube foot and apical muscle preparations from *A. rubens*. **(A,B)** Representative recordings showing that synthetic ArCT causes dose-dependent relaxation of tube foot **(A)** and apical muscle **(B)** preparations that had been stimulated to contract by application of 10 μM acetylcholine (ACh). **(C,D)** Graphs showing the dose-dependent relaxing effect of synthetic ArCT on tube foot **(C)** and apical muscle **(D)** preparations. Mean values ± standard deviation were determined from three experiments, using preparations from different individuals. Relaxing activity was quantified as the percentage reversal of contraction induced by application of 10 μM ACh.

*In vitro* bioassays with synthetic ArCT revealed that it has no effect on the contractile state of cardiac stomach preparations (data not shown) but it causes dose-dependent reversal of ACh (10 μM) induced contraction of both tube foot and apical muscle preparations from *A. rubens* (Figure 11). For tube foot preparations, synthetic ArCT caused dose-dependent relaxation, with 74.6 ± 5.2% reversal of ACh-induced contraction observed at the highest concentration tested (10^−5^ M) (Figures 11A,C). For apical muscle preparations, synthetic ArCT caused dose-dependent relaxation, with 40.5 ± 3.8% reversal of ACh-induced contraction observed at the highest concentration tested (10^−6^ M) (Figures 11B,D).

### Identification of a muscle relaxant in the starfish *Patiria pectinifera* that is a calcitonin-type peptide

**Figure 12:**
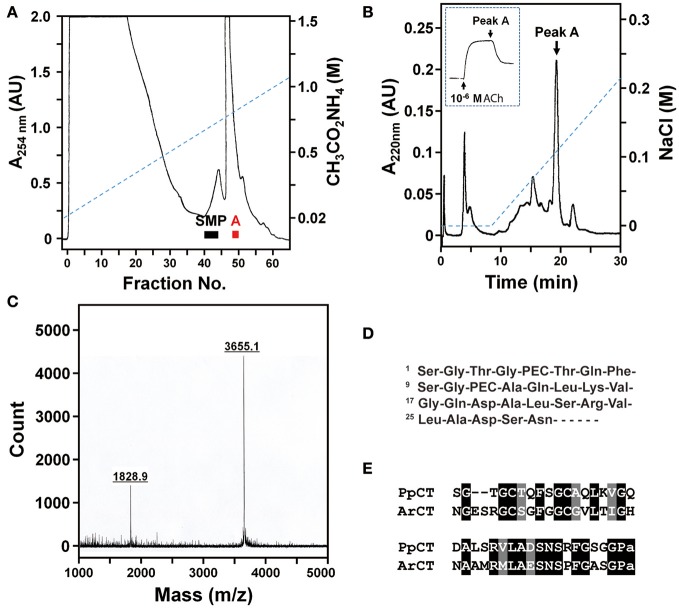
Purification and identification of a muscle relaxant from the starfish *P. pectinifera* that is a calcitonin-type peptide. **(A)** Fractionation of an extract of *P. pectinifera* by cation-exchange chromatography using a linear gradient of 0.02 to 1.5 M ammonium acetate (pH 5.0) at a flow rate of 2.75 ml/min over 6 h. Fractions 40–45 and fractions 49 and 50 exhibited relaxing activity on apical muscle preparations. The bioactive constituent of fractions 40–45 was identified previously as the pedal peptide-type neuropeptide SMP (Kim et al., 2016). The bioactive constituent of fractions 49 and 50, designated “Peak A,” was purified here. **(B)** Following several purification steps, cation-exchange HPLC (0–0.22M NaCl over 22 min) was used here to obtain a single absorbance peak containing bioactive peak A. The inset shows that an aliquot of purified peak A causes relaxation of an apical muscle preparation from *P. pectinifera* that had been contracted with 10^−6^ M ACh. **(C)** Analysis of peak A using MALDI TOF MS reveals that doubly and singly charged ions have molecular masses of 1828.9 and 3655.1 Da, respectively. **(D)** Partial sequence of purified peak A, where PEC represents modified cysteine caused by reduction and alkylation. **(E)** Determination of the sequence of peak A based upon mass spectral analysis and sequencing of the purified peptide and sequencing of a cDNA encoding its precursor (see supplementary Figure 3) reveals that it is a calcitonin-type peptide (PpCT) that shares sequence similarity with ArCT.

Previously, we reported the identification of starfish myorelaxant peptide (SMP), a muscle relaxant in the starfish *P. pectinifera* that is a pedal peptide/orcokinin-type neuropeptide (Kim et al., 2016). Here we sought to determine the molecular identity of a second muscle relaxant in *P. pectinifera* (peak A) that elutes later than SMP when extracts of *P. pectinifera* are fractionated by cation-exchange column chromatography (Figure 12A). Purification of peak A was accomplished in four chromatographic steps, which sequentially were two RP, anion-exchange, and cation-exchange HPLC. A single absorbance peak (peak A) was obtained from the cation-exchange HPLC and an aliquot of this peak caused relaxation of the apical muscle from *P. pectinifera* (Figure 12B). Using MALDI TOF MS, the molecular mass of peak A was determined to be 1828.9 or 3655.1 Da as doubly or singly charged ions, respectively (Figure 12C). Following reduction and alkylation, automated Edman degradation was used to determine the N-terminal sequence of the purified peptide. The first 29 residues of peak A were identified as Ser-Gly-Thr-Gly-PEC-Thr-Gln-Phe-Ser-Gly-PEC-Ala-Gln-Leu-Lys-Val-Gly-Gln-Asp-Ala-Leu-Ser-Arg-Val-Leu-Ala-Asp-Ser-Asn, where pyridylethylated cysteine (PEC) represents a modified cysteine residue through reduction and alkylation (Figure 12D). The monoisotopic mass of this sequence including one disulfide bond between Cys^5^ and Cys^11^ is 2910.4 Da, which is not in accordance with the molecular mass of the native peak A (Figure 12C), indicating that the peptide contains more unidentified residues at the C-terminus. Consistent with this hypothesis, BLAST analysis of the GenBank database revealed that peak A shares a high level of sequence similarity with ArCT, which is C-terminally extended with respect to peak A. Therefore, peak A was named *Patiria pectinifera* calcitonin (PpCT).

### Determination of the sequence of the PpCT precursor

To determine the sequence of the PpCT precursor (PpCTP), a cDNA encoding PpCTP was cloned using RACE-PCR with degenerate primers based on the amino acid sequence of the purified peptide. A 929 bp cDNA encoding PpCTP was sequenced and found to comprise a 117 bp 5′-untranslated region (UTR), a 366 bp open reading frame (ORF) and a 446 bp 3′-UTR including a poly-A tail (Supplementary Figure 3). The ORF of PpCTP is a 121-residue protein, comprising a 21-residue signal peptide (as predicted by SignalP; v4.1; http://www.cbs.dtu.dk/services/SignalP/) and a 38-residue CT-type peptide (including a C-terminal glycine residue) flanked by endoproteolytic stites (K^68^R^69^ and K^108^R^109^) (Supplementary Figure 3). Based on the sequence of PpCTP, the theoretical molecular monoisotopic mass of the mature PpCT peptide with a disulfide bond between Cys^5^ and Cys^11^ and C-terminal amidation is 3654.7 Da (http://www.peptidesynthetics.co.uk/tools/), which is consistent with the experimentally obtained molecular mass of peak A (3655.1 Da). Therefore, the structure of PpCT is SGTG*C*TQFSG*C*AQLKVGQDALSRVLADSNSRFGSGP-NH_2_, with a disulphide bridge between the underlined cysteine residues. An alignment of the sequences of PpCT and ArCT is shown in Figure 12E.

### Relative expression levels of PpCTP and bioactivity of PpCT in *P. pectinifera*

**Figure 13:**
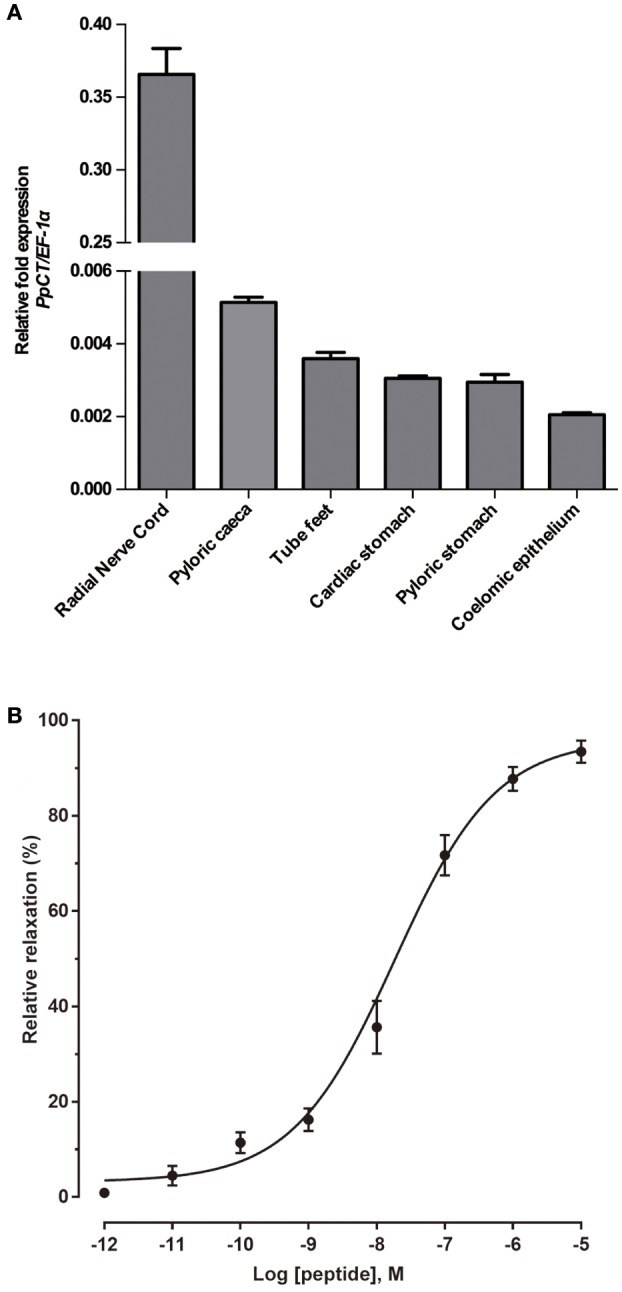
Quantitative analysis of the expression levels of the PpCT precursor transcript in *P. pectinifera* organs/tissues and pharmacological analysis of the relaxing effect of synthetic PpCT on apical muscle preparations. **(A)** Analysis of the expression of the PpCTP transcript using RT-qPCR reveals that the highest expression level is in radial nerve cords. Expression was also detected in other organs/tissues analyzed but at a level that is two orders of magnitude lower than in radial nerve cords. Mean values with standard deviations (*n* = 3) are shown. **(B)** Synthetic PpCT causes dose-dependent relaxation of apical muscle preparations from *P. pectinifera*. Mean values ± standard deviation were determined from 5 separate experiments. Relaxing activity was calculated as the percentage reversal of contraction of the apical muscle caused by 10^−6^ M ACh.

Analysis of the relative expression levels of the PpCTP mRNA using RT-qPCR revealed the highest expression in radial nerve cords. Expression of PpCTP was also detected in pyloric caeca, tube feet, cardiac stomach, pyloric stomach, and coelomic epithelium, but the expression level in these tissues was two orders of magnitude lower than in the radial nerve cords (Figure 13A). Consistent with the relaxing effect of peak A on *in vitro* apical muscle preparations from *P. pectinifera* (Figure 12B), synthetic PpCT caused dose-dependent relaxation of apical muscle preparations with the threshold effect, *p*EC_50_ and E_max_ at 10^−11^ M, 7.74 ± 0.09 M and 96.0 ± 3.0%, respectively (Figure 13B).

## Discussion

Here we report the first molecular, neuroanatomical and functional characterization of a calcitonin-type peptide (ArCT) in a deuterostomian invertebrate—the starfish *Asterias rubens* (phylum Echinodermata). Comparison of the sequence of ArCT with CT-type peptides from other species reveals conservation of several structural features that are characteristic of this family of neuropeptides, including two N-terminal cysteine residues that form a disulfide bond followed by an amphipathic alpha-helix and C-terminal amidation. Furthermore, comparative analysis of the sequence of the ArCT precursor (ArCTP) with CT-type precursors in other species revealed that, consistent with the phylogenetic position of *A. rubens* as an ambulacrarian deuterostomian invertebrate, ArCTP clusters with other deuterostomian CT-type precursors and is positioned in a clade also comprising CT-type precursors from other ambulacrarians (other echinoderms and hemichordates) that is distinct from a clade comprising CT-type precursors from chordates.

To facilitate functional characterzation of ArCT, it was first essential to determine its molecular structure. Analysis of an extract of radial nerve cords from *A. rubens* using LC-MS-MS confirmed the predicted structure of ArCT—a 39 amino acid residue peptide with an intramolecular disulfide bond between N-terminally located cysteine residues and with an amidated C-terminal proline residue. Having determined the structure of ArCT, we investigated the expression of ArCTP transcripts and ArCT in *A. rubens*. We have previously reported our analysis of the expression of ArCTP transcripts during the larval stages of *A. rubens* using mRNA *in situ* hybridization. No expression of ArCTP was observed during early larval development in the two-armed bipinnariae but by the brachiolaria stage, which has three additional pairs of brachiolar arms, cells expressing ArCTP were observed in the adhesive disk and in adjacent tissue surrounding the disk (Mayorova et al., 2016). The adhesive disk mediates larval attachment to the substratum prior to metamorphosis of the bilaterally symmetrical larval stage into a pentaradially symmetrical juvenile (Haesaerts et al., 2005; Murabe et al., 2007). Therefore, we speculated that ArCT may be involved in regulation of physiological processes associated with larval attachment (Mayorova et al., 2016), but further studies are required to address this issue. Here we extended our analysis of ArCTP expression to the adult stage of *A. rubens*. Furthermore, by generating novel specific antibodies to ArCT, visualization of the distribution of a CT-type peptide has been accomplished in an echinoderm for the first time, as discussed below.

Consistent with the original identification of ArCTP transcripts in the radial nerve cords of *A. rubens* (Semmens et al., 2016), extensive expression of ArCTP and ArCT was revealed in both the radial nerve cords and circumoral nerve ring. Accordingly, analysis of the expression of an ortholog of ArCTP in the starfish *P. pectinifera* (PpCTP) using RT-qPCR revealed the highest level of expression in the radial nerve cords. Interpretation of the physiological significance of ArCT expression in the nervous system of *A. rubens* is to some extent restricted by our limited knowledge of its functional architecture in this species and in other echinoderms (Mashanov et al., 2016). The ectoneural region of the starfish nervous system is thought to comprise a mixture of sensory, interneuronal and motor neurons (Smith, 1950a,b; Cobb, 1987; Mashanov et al., 2016) but it is not possible at present to determine if ArCT-expressing cells belong to one or more of these neuronal classes. Nevertheless, ArCT-expressing cells in the ectoneural region of the nervous system are clearly neurons because of the presence of intense immunostaining in the ectoneural neuropile, which contains the axonal processes of ectoneural neurons. The hyponeural regions of the nervous system are thought to comprise only motor neurons (Smith, 1937; Pentreath and Cobb, 1972) and therefore it can be inferred that ArCT-expressing hyponeural cells are motor neurons. Consistent with this notion, ArCT-containing axonal processes can be seen to project directly from the hyponeural region to innervate the infra-ambulacral muscle and ArCT-immunoreactivity is present in the lateral motor nerves. Branches of the lateral motor nerves project into the coelomic lining of the arms (Smith, 1937, 1950a) and accordingly ArCT-immunoreactive processes can be seen in association with the circular muscle layer of the coelomic lining. Strands of muscle derived from the circular muscle layer of the coelomic lining are attached to ossicles of the body wall and ArCT-immunoreactive fibers are also present in close association with these muscle strands. ArCTP expression and ArCT-immunoreactivity were also revealed in association with other muscular organ systems in *A. rubens*, including several regions of the digestive system, the tube feet (locomotory organs), the apical muscle (which mediates arm flexion), papulae (which enable gas exchange across the body wall) and the gonoduct. These findings suggest that ArCT may be involved in regulation of a variety of physiological or processes in starfish, including feeding, digestion, locomotion, gas exchange and gamete release.

Informed by the patterns of expression of ArCTP and ArCT, we hypothesized that ArCT may act as neural regulator of muscle activity in starfish. To test this hypothesis we examined the effects synthetic ArCT on three *in vitro* preparations of neuromuscular organs from *A. rubens*—the cardiac stomach, tube feet and apical muscle. Our previous studies have revealed that other neuropeptides cause relaxation (e.g., SALMFamides, pedal peptide-type neuropeptides; Melarange et al., 1999; Lin et al., 2017a) or contraction (e.g., NGFFYamide, ArGnRH, ArCRZ; Semmens et al., 2013; Tian et al., 2017) of one or more of these preparations. Interestingly, ArCT did not cause relaxation (or contraction) of cardiac stomach preparations and therefore this peptide may regulate other physiological processes in the cardiac stomach and in other regions of the digestive system. For example, ArCT could be involved in regulation of ciliary mediated transit of food material and/or regulation of the secretion of digestive enzymes. We did, however, observe that ArCT causes dose-dependent relaxation of *A. rubens* tube foot and apical muscle preparations *in vitro*.

In parallel with our investigation of the expression and actions of ArCT in *A. rubens*, we purified a peptide that acts a muscle relaxant in the starfish *P. pectinifera*. Determination of the structure of this peptide revealed that it is a CT-type peptide that shares a high level of sequence similarity with ArCT. Thus, two independent lines of research have revealed that CT-type peptides act as muscle relaxants in starfish. Recent molecular analysis of the phylogenetic relationships of extant starfish (class Asteroidea) indicates that there are two distinct clades—a clade that includes the orders Forcipulatida and Velatida and a clade that includes the orders Paxillosida, Notomyotida, Spinulosida, and Valvitida (Linchangco et al., 2017). It is noteworthy that *A. rubens* (order Forcipulatida) belongs to the first clade and *P. pectinifera* (order Valvitida) belongs to the second clade and therefore CT-type peptides have been shown to act as muscle relaxants in species belonging to two evolutionarily divergent starfish orders. We can infer from this that the action of CT-type peptides as muscle relaxants may be traceable to the common ancestor of all extant starfish. Investigation of the actions of CT-type peptides in a variety of other starfish species belonging to other orders will provide further insight into this issue. CT-type peptide precursors have also been identified in other echinoderms, including brittle stars (class Ophiuroidea) (Zandawala et al., 2017), sea urchins (class Echinoidea) (Rowe and Elphick, 2012), and sea cucumbers (class Holothuroidea) (Rowe et al., 2014). Interestingly, in both sea cucumbers and brittle stars, precursors comprising either one or two copies of CT-type peptides have been identified and, similar to the CT/CGRP gene in vertebrates, this is attributed to alternative splicing of transcripts encoded by the same gene (Rowe et al., 2014; Zandawala et al., 2017; Suwansa-Ard et al., 2018), However, nothing is known about the physiological roles of CT-type peptides in these animals. Informed by the findings of this study, it would be interesting to investigate if CT-type peptides also act as muscle relaxants in other echinoderms.

Our discovery that CT-type peptides act as muscle relaxants in starfish provides a new insight into the evolution of the physiological roles of CT-type peptides. To the best of our knowledge, there have been no reports of CT itself acting as a muscle relaxant in mammals or other vertebrates. However, α-CGRP, which is encoded by the same gene as CT, is probably best known for its actions as a potent and powerful vasodilator in mammals (Brain et al., 1985) and in other vertebrates (Kagstrom and Holmgren, 1998; Shahbazi et al., 2009). Furthermore, CGRP also causes relaxation of intestinal longitudinal muscle and inhibition of intestinal peristalsis in mammals (Holzer et al., 1989) and, accordingly, CGRP inhibits gut motility in fish (Shahbazi et al., 1998). Thus, the action of CGRP as a smooth muscle relaxant appears to be an evolutionarily ancient function of this neuropeptide in vertebrates. Furthermore, CGRP is also synthesized and released by skeletal motoneurons in vertebrates and causes down-regulation of acetylcholinesterase expression in muscle fibers (Rossi et al., 2003).

**Figure 14:**
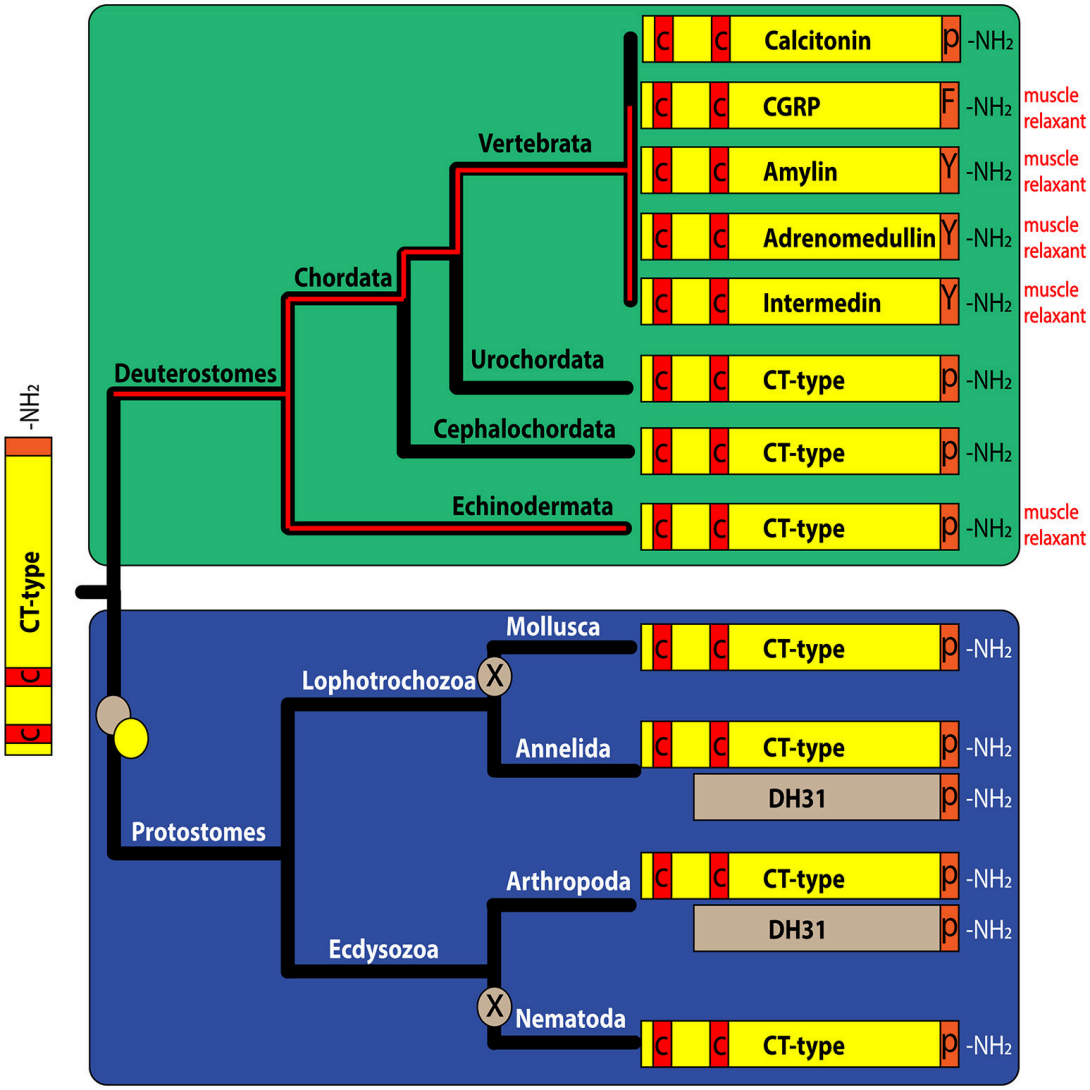
Schematic showing the phylogenetic distribution and structural properties of calcitonin-related peptides in the Bilateria and the occurrence of calcitonin-related peptides that have been shown to act as muscle relaxants. Calcitonin and at least five other calcitonin-related peptides typically occur in vertebrate species, whereas in deuterostomian invertebrates, there is typically only a single CT-type peptide. Several calcitonin-related peptides act as muscle relaxants in vertebrates. Our discovery that CT-type peptides act as muscle relaxants in starfish (phylum Echinodermata) indicates that the evolutionary origin of this physiological role can be traced back to the common ancestor of the deuterostomes. In the protostomian lineage gene duplication has given rise to two types of calcitonin-related peptides—CT-like peptides with an N-terminal disulfide bond and DH31-type that lack an N-terminal disulfide bond. In protostomian invertebrates, the physiological roles of DH31-type peptides have been characterized (see discussion) but nothing is known about the physiological roles of CT-like peptides with an N-terminal disulfide bond.

Interestingly, the actions of CGRP as a regulator of muscle activity in vertebrates is not unique to this member of the family of CT-related peptides in vertebrates. Other CT-related peptides also act as vascular smooth muscle relaxants, including amylin, adrenomedullin and intermedin (adrenomedullin 2) (Steiner et al., 1991; Pinto et al., 1996; Yoshimoto et al., 1998; Edvinsson et al., 2001; Golpon et al., 2001; Kandilci et al., 2008). The occurrence of multiple genes encoding CT-related peptides in vertebrates contrasts with invertebrate chordates. Thus, in both the urochordate *Ciona intestinalis* and the cephalochordate *Branchiostoma floridae* there is a single gene encoding a CT-type peptide precursor (Sekiguchi et al., 2009, 2016). The existence in vertebrate species of multiple genes encoding CT-related peptides is thought to be a consequence of genome duplications as well as local genic or intragenic duplication events (Chang et al., 2004; Ogoshi et al., 2006). Because several members of the family of CT-related peptides act as smooth muscle relaxants, it seems likely therefore that this is an evolutionarily ancient role that would also have been a characteristic of the common molecular ancestor of CT-related peptides in vertebrates. With our discovery that CT-type peptides in starfish (*A. rubens* and *P. pectinifera*) act as muscle relaxants, we propose that the evolutionary origin of this physiological role can be traced further back in animal phylogeny to the common ancestor of the deuterostomes (Figure 14).

Can the evolutionary history of CT-related peptides acting as muscle relaxants be pushed back further still to the common ancestor of the Bilateria? To address this issue it will be necessary to investigate the actions of CT-related peptides in protostomian invertebrates. A complicating factor here is that in the protostomian lineage there are two types of CT-related peptides: (i) CT-type peptides that are structurally similar to the CT-type peptides in deuterostomes in having an N-terminal pair of cysteine residues and (ii) DH31-type peptides that lack the N-terminal cysteines. The occurrence of these two types of CT-related peptides is thought to be a consequence of gene duplication in a common ancestor of the protostomes (Conzelmann et al., 2013). In some protostomes both of the two types of CT-related peptides have been retained, whist in some lineages only one type has been retained (Figure 14) (Conzelmann et al., 2013; Veenstra, 2014). Investigation of the physiological roles of the DH31-type peptides has revealed that, in addition to their actions as diuretic hormones in arthropods (Furuya et al., 2000; Coast et al., 2001), they are also involved in regulation of other physiological processes. For example, DH31 affects gut motility in insects and triggers initiation of pre-ecdysis motor activity in the moth *Manduca sexta* (Kim et al., 2006; Brugge et al., 2008). Furthermore, DH31 also acts as a circadian wake-promoting signal in *Drosophila melanogaster* (Kunst et al., 2014). In contrast to the growing body of literature on DH31 function in arthropods, currently nothing is known about the physiological roles of CT-type peptides with an N-terminal pair of cysteine residues in arthropods and other protostomes. This probably reflects the fact that these were discovered only relatively recently from analysis of genome sequence data (Mirabeau and Joly, 2013; Veenstra, 2014) and the loss of these CT-type peptides in some lineages, including the model insect *D. melanogaster*. CT-type peptides with an N-terminal pair of cysteines are, however, present in nematodes, including *C. elegans*. Therefore, there remain exciting opportunities ahead to investigate the physiological roles of CT-type peptides with an N-terminal pair of cysteines in protostomes and to compare with findings from vertebrates and our findings from a deuterostomian invertebrate, as reported here.

## Ethics statement

Approval by the local institution/ethics committee was not required for this work because experimental work on starfish is not subject to regulation.

## Author contributions

WC, ME, and MRE carried out the analysis of the expression of ArCTP and ArCT using mRNA *in situ* hybridization and immunohistochemistry. WC carried out the sequence analysis of ArCT and ArCTP and examination of the *in vitro* pharmacological effects of ArCT. CZ and AJ carried out the structural characterization of ArCT using mass spectrometry. C-HK, H-JG, and NGP performed the purification and structural characterization of PpCT, analysis of the expression PpCTP and analysis of the *in vitro* pharmacological effects of PpCT. The paper was written by WC, NGP, and MRE, with contributions from other authors. The study was conceived and designed by NGP and MRE. All authors gave final approval for publication.

### Conflict of interest statement

The authors declare that the research was conducted in the absence of any commercial or financial relationships that could be construed as a potential conflict of interest.
