# Supplementary material for: Biochemical, Anatomical, and Pharmacological Characterization of Calcitonin-Type Neuropeptides in Starfish: Discovery of an Ancient Role as Muscle Relaxants
Source: Front Neurosci. 2018 Jun 8;12:382. doi: 10.3389/fnins.2018.00382 (PMC6002491; doi:10.3389/fnins.2018.00382)
Supplement: Supplementary file 1 [file Data_Sheet_1.DOCX]

Supplementary Material

**Biochemical, anatomical and pharmacological characterization of calcitonin-type neuropeptides in starfish: discovery of an ancient role as muscle relaxants**

**Weigang Cai, Chan-Hee Kim, Hye-Jin Go, Michaela Egertová, Cleidiane G. Zampronio, Alexandra M. Jones, Nam Gyu Park and Maurice R. Elphick**

# 1. Supplementary Figures and Tables

**1.1 Supplementary Tables**

**Supplemental Table 1: Accession numbers and/or citations for the sequences in Figure 1.**

| **ABBREVIATION** | **SPECIES NAMES** | **ACCESSION NUMBERS AND/OR CITATIONS** |
| --- | --- | --- |
| A.japCT1/CT2 | *Apostichopus japonicus* | Rowe et al. (2014) |
| A.rubCT | *Asterias rubens* | GenBank: KT601715.1 |
| B.floCT1 | *Branchiostome floridae* | GI:260826569 |
| B.floCT2 | *Branchiostome floridae* | GI:260826567 |
| B.floCT3 | *Branchiostome floridae* | GI:260826573 |
| C.eleCT | *Caenorhabditis elegans* | Rowe et al. (2014) |
| C.intCT | *Ciona intestinalis* | GenBank: BAI63095.1 |
| C.telCT | *Capitella telata* | GI: 161220966 |
| C.telDH31 | *Capitella telata* | Rowe et al. (2014) |
| D.melDH31 | *Drosophila melanogaster* | NP_523514.1 |
| D.punDH31 | *Diploptera punctata* | P82372.1 |
| H.sapCT | *Homo sapiens* | GI:179819 |
| H.sapCGRP | *Homo sapiens* | GI:269784661 |
| L.gigCT | *Lottia gigantea* | GI:163526287 |
| L.migCT | *Locusta migratoria* | Veenstra. (2014) |
| L.migDH31 | *Locusta migratoria* | GenBank: AKN21237.1 |
| S.kowCT1 | *Saccoglossus kowalevskii* | Rowe et al. (2014) |
| S.kowCT2 | *Saccoglossus kowalevskii* | GI:187217193 |
| S.purCT | *Strongylocentrotus purpuratus* | GI:115767208 |
| T.rubCT | *Takifugu rubripes* | GI: 19772949 |
| T.rubCGRP | *Takifugu rubripes* | GI: 19772948 |

**Supplemental Table 2: Primers used for RACE-PCR and RT-qPCR analysis of PpCT precursor expression in *P. pectinifera***

| Primers | Sequence (5’-3’) | Used |
| --- | --- | --- |
| PpCT-deg1F | ACNCARTTYWSNGGNMGNGCNCA | 3’RACE |
| PpCT-deg2F | CARYTNAARGTNGGNCARGAYGC | 3’nested RACE |
| PpCT-gsp1R | CTACGCCTTGTCCTCAGCTG | 5’RACE |
| PpCT-gsp2R | GTGTCCACTGATCGCCTTCTC | 5’nested RACE |
| PpCT qPCR-F | CGTAGACGGGATGCACTTCA | RT-qPCR |
| PpCT qPCR-R | GACAACAAACATGGCGGGAG |  |
| EF-1α qPCR-F | TCAACGACTACCAGCCCCTA | RT-qPCR |
| EF-1α qPCR-R | TTCTTGCTAGCCTTCTGGGC |  |

**1.2 Supplementary Figures**

**Supplementary Figure 1:** **Mass spectrometric identification of ArCT in extracts of *A. rubens* radial nerve cords.** (a) MS/MS data for ArCT derived from radial nerve cord extract under native conditions. The b series of the peptide fragment ions are shown in red, the y series in blue and additional peptide fragment ions in green. The m/z of the precursor ion was 949.18 with charge state 4 +. Collectively, these data are consistent with peptide structure shown, including a disulfide bridge between the two cysteine residues and C-terminal amidation. (b) MS/MS data for ArCT derived from radial nerve cord extract that was subject to reduction and alkylation but without tryptic digestion. The m/z of the precursor ion was 978.20 with charge state 4 +. Collectively, these data are consistent with peptide structure shown, with C-terminal amidation but without a disulfide bridge between the two cysteine residues. (c) MS/MS data for a fragment of ArCT derived from radial nerve cord extract that was subjected to tryptic digestion without reduction and alkylation. The m/z of the precursor ion was 635.96 with charge state 3 +. Collectively, these data are consistent with peptide structure shown, including a disulfide bridge between the two cysteine residues. (d) MS/MS data for a fragment of ArCT derived from radial nerve cord extract that was subjected to tryptic digestion without reduction and alkylation. The m/z of the precursor ion was 682.32 with charge state 2 +. Collectively, these data are consistent with peptide structure shown, including C-terminal amidation, which provides an additional basic group amenable to protonation.

**Supplementary Figure 2:** **Characterization of a rabbit antiserum to ArCT using an enzyme-linked immunosorbent assay (ELISA).** (a): Incubation of antiserum (red) and pre-immune serum (blue) at dilutions between 1:500 to 1:128000 with 0.1 nmol of antigen peptide (ArCT-ag) per well reveals that absorbance values with the pre-immune serum are indistinguishable from a blank control without serum (black), whereas with the antiserum immunoreaction is observed with dilutions ranging from 1:500 to 1:32,000. (b) Incubation of antiserum (red) and pre-immune serum (blue) at a dilution of 1:16,000 with between 1 × 10^-16^ and 1 × 10^-9^ moles of antigen peptide (ArCT-ag) per well reveals that absorbance values with the pre-immune serum are indistinguishable from a blank control without serum (black), whereas with the antiserum the antigen is detected at dilutions ranging from 1 × 10^-13^ to 1 x 10^-9^ moles per well. Data points are mean values determined from two separate experiments, each performed in duplicate.

1 catttcaagtgcagtaaaacgcggggaaagtacggaacctcaccttccgtcgctcacagc

61 cggaaaacagctgacgggatatcttctgaaagatcactgacgtcatcaatcgcagccatg

**M** 1

121 aagccaacagtagctctaacgctagcagttctgtgcacattctacaccatcgccacagcc

**K P T V A L T L A V L C T F Y T I A T A** 21

181 gcctcattagcaagagccgaagcagaccttatgttcccactgacaggagacgatttgaga

**A S L A R A E A D L M F P L T G D D L R** 41

241 gaattagcagacaaagtcgacgcatacgacgaaatcctactcatgttttccggtagcagt

**E L A D K V D A Y D E I L L M F S G S S** 61

301 gaattccagtcaatgctaaaaagatcaggcactggttgtacacaattcagcggctgcgca

**E F Q S M L K R S G T G C T Q F S G C A** 81

361 caactgaaggtcggacaggatgctctatcgcgcgtactcgcagacagcaattcccgcttc

**Q L K V G Q D A L S R V L A D S N S R F** 101

421 gggagtggtgggccaggaaagagaaggcgatcagtggacacaccagctgaggacaaggcg

**G S G G P G K R R R S V D T P A E D K A** 121

481 tagacgggatgcacttcaaaagtagtccaggacgatgcattataggcttggacatccaca

*****

514 agattgcttccaccgcatgcacacactacacgcacgtccgaatggccaagttttgactct

601 cccgccatgtttgttgtcaagagcacagcgccctctacaacttgtttgacttccagccaa

661 tagagcaccatattgactgcccgaaaattataatgtggttgggatgtaatctgatctggc

721 gctagtttagttggcaacaaatatggcggtatgcacttgcgttttagaggctaatgactg

781 tgtaagctataaccaaaaaacaaaaaaaaatccatcttggtcaacctgataaagcaccag

841 catataatttgcttcaaaactatgttgaacaaaaaacaagacggtttatgataaatcagt

901 aattacaacactaaaaaaaaaaaaaaaaa

**Supplementary Figure 3: Sequence of a cDNA encoding the *Patiria pectinifera* calcitonin-type precursor (PpCTP).** The nucleotide sequence (lowercase, 929 bases) of a cDNA encoding PpCTP (uppercase, 121 amino acid residues) is shown. Primers used for cloning are represented in bold and underlined text. The predicted signal peptide is shown in blue, the calcitonin-type peptide PpCT (with cysteine residues underlined) is shown in red, including a C-terminal glycine residue that is a substrate for amidation (orange). Dibasic cleavage sites are shown in green. The asterisk shows the position of the stop codon.
